# CSGDA: a cell state-guided graph domain adaptation network for single-cell drug response prediction

**DOI:** 10.1093/bioinformatics/btag726

**Published:** 2026-09-29

**Authors:** Fen Yan, Donggui Xie, Xiyue Cao, Feiqiao Mao, Zhuhong You, Yitao Chen, Zhihua Du, Yu-An Huang

**Affiliations:** College of Computer Science and Software Engineering, Shenzhen University, Shenzhen, Guangdong 518000, China; College of Computer Science and Software Engineering, Shenzhen University, Shenzhen, Guangdong 518000, China; School of Computer Science, Northwestern Polytechnical University, Xi’an, Shaanxi 710000, China; College of Computer Science and Software Engineering, Shenzhen University, Shenzhen, Guangdong 518000, China; School of Computer Science, Northwestern Polytechnical University, Xi’an, Shaanxi 710000, China; College of Computer Science and Software Engineering, Shenzhen University, Shenzhen, Guangdong 518000, China; College of Computer Science and Software Engineering, Shenzhen University, Shenzhen, Guangdong 518000, China; School of Computer Science, Northwestern Polytechnical University, Xi’an, Shaanxi 710000, China

## Abstract

**Motivation:**

Intratumoral heterogeneity drives cancer recurrence and metastasis, yet single-cell drug response prediction faces severe “cross-domain” challenges, such as applying *in vitro* models to *in vivo* tissues or inferring metastatic resistance from primary tumors. These scenarios trigger distribution shifts arising from heterogeneous sequencing platforms, distinct tissue microenvironments, and metastatic evolution—problems rarely addressed by existing methods.

**Results:**

We introduce CSGDA, a cell state-guided graph domain adaptation framework designed to predict drug responses across these biological heterogeneities. CSGDA incorporates biological priors to map gene expression into functional cell states, guiding a structure-learning module to construct robust cell topology. To mitigate distribution shifts, the model employs graph domain adaptation combined with a novel overlap penalty mechanism. Extensive benchmarks on five scRNA-seq datasets demonstrate that CSGDA outperforms the state-of-the-art method SSDA4Drug by approximately 6 percentage points in both ACC and AUPR. Beyond prediction accuracy, we employed integrated gradients to effectively pinpoint key genes involved in drug resistance within a challenging cross-metastasis cisplatin dataset. These findings underscore CSGDA’s superior performance in single-cell drug response prediction and its potential in resolving single-cell heterogeneity, paving the way for precision medicine.

**Availability and implementation:**

Source code is available at https://github.com/yanfen-git/CSGDA-code and archived at https://doi.org/10.5281/zenodo.21759274. Data are available at https://doi.org/10.5281/zenodo.21770737.

## 1 Introduction

Intratumoral heterogeneity constitutes the molecular basis of drug resistance and recurrence (Marusyk *et al.* 2020, Ghorbian 2025), yet the homogenized profiles derived from traditional Bulk RNA-seq inevitably mask critical rare subpopulations. Although scRNA-seq offers an unprecedented single-cell perspective for unraveling tumor microenvironment evolution and resistance mechanisms (Huang *et al.* 2023), reliance solely on wet-lab experiments to construct comprehensive drug response atlases lacks economic viability and scalability in clinical implementation due to prohibitive costs and complex protocols (Van de Sande *et al.* 2023).

Existing methods for predicting drug responses, such as SCAD (Zheng *et al.* 2023), scDEAL (Chen *et al.* 2022), scAdaDrug (Liu *et al.* 2025), SSDA4Drug (Huang and Liu 2025), and scGSDR (Huang *et al.* 2025), primarily adhere to a “Bulk-to-Single-Cell” cross-modal transfer learning paradigm, utilizing large-scale cell line population data as the source domain to transfer drug response knowledge to single-cell data (Maeser *et al.* 2024, Yan *et al.* 2025). However, this paradigm faces dual challenges in real-world scenarios. The first is Inherent Manifold Structure Mismatch. From a geometric perspective, Bulk data converges to the “centroid of a convex hull,” representing smoothed population averages, whereas scRNA-seq data constitutes highly non-linear “non-convex manifolds.” Forcing the alignment of such topological heterogeneity often results in subtle drug response features being overshadowed by statistically significant batch effects or broad cell-type main effects (Hui *et al.* 2024). The second is complex non-I.I.D. shifts. Multi-level distribution deviations introduced by discrepancies in sequencing platforms and microenvironments severely compromise the stability of feature alignment, leading to decision boundary drift (Park *et al.* 2021). Consequently, existing methodologies are largely confined to idealized or homologous contexts, failing to incorporate highly heterogeneous real-world clinical scenarios, such as cross-tissue adaptation and cross-metastasis evolution, which are essential for modeling the true complexities of actual patient data(Gundem *et al.* 2015, Savino *et al.* 2025).

To address these challenges, we propose CSGDA, a cell-state-guided graph domain adaptation framework for drug response prediction under the direct SC-to-SC transfer paradigm. CSGDA uses CancerSEA-derived (Yuan *et al.* 2019) functional states as semantic anchors to construct biologically meaningful cell–cell topologies and align topology-aware representations across heterogeneous single-cell domains without target response labels. The main contributions of this study are summarized as follows:

1. To our knowledge, CSGDA is the first graph domain adaptation framework to explicitly incorporate CancerSEA functional states as biological priors for adaptive graph structure learning in cross-domain single-cell drug response prediction. The proposed Cell State-Aware Graph Structure Learning (CSA-GSL) module refines cell–cell topology under low-rank, sparsity, and feature-smoothness constraints, thereby reducing spurious connections induced by scRNA-seq dropout noise.
2. CSGDA performs domain adaptation on graph-encoded representations under the SC-to-SC transfer paradigm without using target response labels. A shared Graph Transformer extracts topology-aware representations, which are aligned through adversarial domain adaptation and target entropy minimization. A joint cross-entropy and overlap-penalty objective further improves the separation between sensitive and resistant cells under heterogeneous domain shifts.
3. CSGDA is systematically evaluated on five SC-to-SC tasks, outperforming representative state-of-the-art methods. Integrated Gradients (IGs) analysis further identifies high-contribution resistance genes and biologically coherent functional programs, demonstrating the predictive robustness and biological interpretability of the framework.

## 2 Materials and methods

As shown in Fig. 1, CSGDA is an unsupervised SC-to-SC graph domain adaptation framework for cross-domain single-cell drug response prediction. It consists of three core steps: (i) refining cell graphs of both domains via a CSA-GSL module with CancerSEA priors; (ii) encoding topology-aware representations with a shared Graph Transformer; and (iii) aligning cross-domain features via adversarial adaptation and target entropy minimization, with a hybrid cross-entropy and overlap-penalty loss for source classification. No target response labels are used during training.

**Figure 1:**
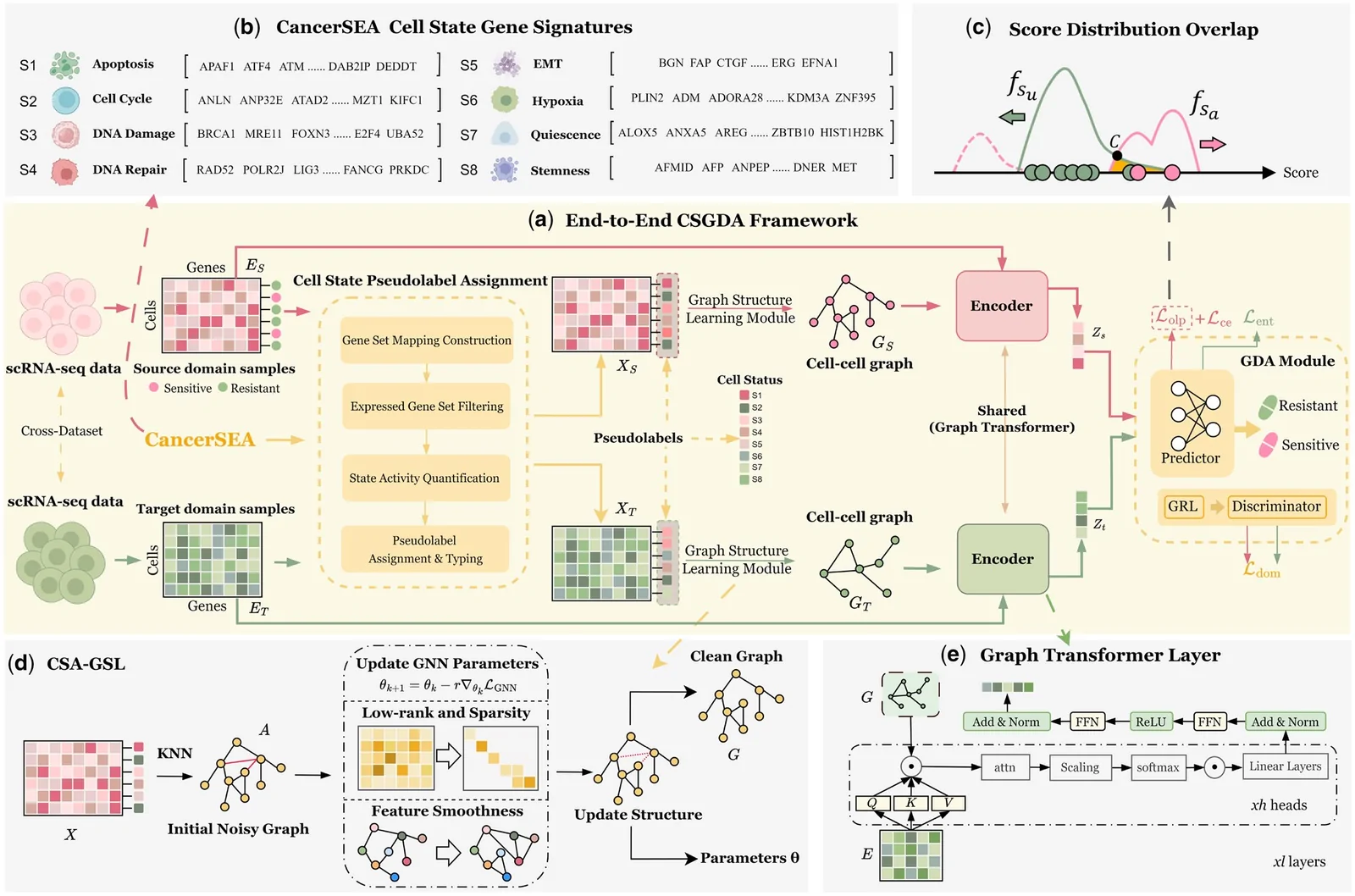
The architecture of CSGDA. The framework comprises (a) the complete End-to-End CSGDA framework for cross-domain drug response prediction, (b) biological prior construction, (c) the GDA module incorporating an overlap penalty mechanism, (d) the CSA-GSL module for robust topology, and (e) the shared graph transformer encoder.

### 2.1 Cell state-guided graph structure learning module

To address spurious connections in cell graphs caused by scRNA-seq dropout noise, we propose the CSA-GSL module (Fig. 1d) to reconstruct biologically meaningful intercellular topology, which builds an initial noisy graph with biological priors and denoises it via a ProGNN-based optimization framework.

#### 2.1.1 Cell state extraction and pseudo-labeling

As depicted in Fig. 1b, we first construct a state-gene mapping from the CancerSEA database, selecting 8 drug resistance-related functional states as biological anchors. For each cell, we compute the activity score of each state by averaging the expression of overlapping marker genes, yielding an 8-dimensional cell state feature matrix $X\in R^{N\times 8}$ that mitigates dropout effects:


(1) $$x_{i,k}=\frac{1}{\left|G_{k}\cap G_{\text{data}}\right|}\sum_{g\in (G_{k}\cap G_{\text{data}})}E_{i,g}$$

Here, $x_{i,k}$ denotes the state activity score of cell *i* for the *k*-th state, where $E_{i,g}$ represents the expression level of gene *g* in cell *i*. $G_{k}$ and $G_{\text{data}}$ correspond to the marker gene set for the *k*-th state and the total gene set in the scRNA-seq dataset, respectively, with $\left|G_{k}\cap G_{\text{data}}\right|$ indicating the count of valid overlapping marker genes.

Pseudo-label assignment. Pseudo-labels are assigned via the “Winner-Takes-All” rule, where each cell is labeled with its dominant functional state to guide subsequent graph learning:


(2) $$y_{\text{pseudo}}^{\left(i\right)}=\underset{k\in \{1,\ldots ,8\}}{\text{argmax}}(x_{i,1},x_{i,2},\ldots ,x_{i,8})$$

Here, $y_{\text{pseudo}}^{\left(i\right)}$ denotes the assigned dominant state pseudo-label for cell *i*. The search space {1,…,8} corresponds to the set of eight core functional cell states defined in our framework.

#### 2.1.2 CSA-GSL module

We initialize the cell adjacency graph via KNN similarity on cell-state embeddings, then optimize graph structure G and GNN parameters θ alternately following the alternating optimization paradigm of ProGNN (Jin *et al.* 2020). The unified graph structure learning loss is defined as:


(3) $$L_{\text{GSL}}(G,\theta )=\underset{L_{\text{GNN}}}{\underset{︸}{\gamma L_{\text{GNN}}(\theta ,G,X,Y_{L})}}+\underset{L_{\text{Fid}}}{\underset{︸}{\phi \|G-A{\|}_{F}^{2}}}\;+\underset{L_{\text{Reg}}}{\underset{︸}{\alpha \|G{\|}_{1}+\beta \|G{\|}_{*}+\lambda \cdot \mathrm{tr}(X^{T}{\hat{L}}_{G}X)}}$$

Here, *X* is the cell-state score matrix and $Y_{L}$ denotes the expression-derived state pseudo labels. The hyperparameter γ weights the classification loss against the structural constraints. The hyperparameter ϕ balances reconstruction freedom against prior topology to preserve critical neighborhoods during denoising. Graph refinement incorporates a sparsity constraint $\alpha \|G{\|}_{1}$ ($\alpha =5\times 10^{-4}$) and a low-rank constraint $\beta \|G{\|}_{*}$ (β=1.5). Crucially, we introduce a feature smoothness constraint $\lambda \mathrm{tr}(X^{T}{\hat{L}}_{G}X)$ (λ=0.1) based on the homophily assumption of biological networks, where cells with similar biological states tend to connect.

By employing the normalized graph Laplacian ${\hat{L}}_{G}$, this constraint penalizes edges between cells that exhibit significant differences in their 8-dimensional state features *X*. This mechanism effectively filters out spurious cross-subpopulation associations while mitigating the impact of varying cellular connectivities on feature smoothness. The normalized graph Laplacian ${\hat{L}}_{G}$ is defined based on the degree matrix $D\in R^{N\times N}$ of the clean graph *G*. Here, *D* is a diagonal matrix where each diagonal element represents the number of adjacent cells, corresponding to the cellular connectivity within the single-cell microenvironment:


(4) $$D_{ii}=\sum_{j=1}^{N}G_{ij},\;{\hat{L}}_{G}=D^{-1/2}(D-G)D^{-1/2}$$

CSA-GSL alternately updates G and θ until convergence, and the resulting graph is used by the shared Graph Transformer encoder. Detailed graph construction, regularization, and optimization procedures are provided in Section G.1, available as supplementary data at *Bioinformatics* online.

### 2.2 Shared graph transformer encoder

To extract transferable cell representations, we adopt a weight-shared Graph Transformer encoder, $E_{\text{sh}}$, for the source and target domains. As illustrated in Fig. 1e, the encoder takes the refined cell graphs and gene expression matrices produced by CSA-GSL as inputs and generates topology-aware latent embeddings through graph-constrained multi-head attention:


(5) $$Z_{s}=E_{\text{sh}}(E_{S},G_{S}),\;Z_{t}=E_{\text{sh}}(E_{T},G_{T}),$$

where $E_{S}$ and $E_{T}$ are the source and target expression matrices, $G_{S}$ and $G_{T}$ are their refined graphs, and $Z_{s}\in R^{N_{s}\times d_{h}}$ and $Z_{t}\in R^{N_{t}\times d_{h}}$ are the resulting latent embeddings. Weight sharing applies the same feature mapping to both domains and places their embeddings in a common latent space for subsequent adversarial alignment. Feature projection, graph-constrained multi-head attention, residual normalization, and full-batch processing are detailed in Section G.2, available as supplementary data at *Bioinformatics* online.

### 2.3 Graph-based domain adaptation module

To transfer predictive efficacy from the source domain to an unlabeled target domain, we developed a GDA module rooted in adversarial learning (Fig. 1a). Furthermore, to address class overlap, we employ a specific penalty mechanism (Fig. 1c). This module mitigates distribution shifts arising from sequencing platform variability or tissue heterogeneity. Target expression profiles and expression-derived CancerSEA state pseudo labels are used to construct and refine the target graph. During adaptation, target graph embeddings participate in adversarial alignment, while their predicted class probabilities are used for entropy minimization. Target drug response labels are excluded from these procedures and used only for final evaluation.

#### 2.3.1 Drug response predictor

Given cell embedding $z_{i}$, the predictor generates the class probability distribution ${\hat{y}}_{i}$ (sensitive versus resistant) using a linear projection and Softmax function.


(6) $${\hat{y}}_{i}=\text{softmax}(W_{c}z_{i}+b_{c})$$

#### 2.3.2 Adversarial domain alignment

To reduce the distribution discrepancy between the source and target domains, we employ a domain discriminator *D* to distinguish their graph-encoded representations, while the shared encoder learns representations that confuse the discriminator. The adversarial domain loss is defined as


(7) $$L_{\text{dom}}=-E_{z_{s}∼Z_{s}}\left[\text{log}\;D(z_{s})\right]-E_{z_{t}∼Z_{t}}\left[\text{log}\;\left(1-D(z_{t})\right)\right],$$

where $z_{s}$ and $z_{t}$ denote source and target cell embeddings sampled from $Z_{s}$ and $Z_{t}$, respectively. The discriminator output D(z) represents the probability that an embedding belongs to the source domain. During training, the discriminator minimizes $L_{\text{dom}}$, whereas a Gradient Reversal Layer (GRL) reverses the domain-loss gradient passed to the shared encoder, encouraging domain confusion. The detailed GRL operation and encoder update are provided in Section G.3, available as supplementary data at *Bioinformatics* online.

#### 2.3.3 Target domain information entropy minimization

To enforce the cluster assumption, we minimize the entropy of target probabilities $P_{t}=\text{softmax}(C(Z_{t}))$:


(8) $$L_{\text{ent}}=-\frac{1}{N_{t}}\sum_{i=1}^{N_{t}}\sum_{k=1}^{K}P_{ik}\;\text{log}(P_{ik})$$

Here, $N_{t}$ denotes the target cell count, and $P_{ik}$ represents the probability of cell *i* belonging to class *k* (where K=2). This constraint drives decision boundaries away from high-density data regions.

#### 2.3.4 Classification optimization based on overlap penalty

To enlarge inter-class margins and improve robustness to domain shifts, we propose an overlap penalty loss $L_{\text{olp}}$. We split source prediction probabilities into sensitive and resistant sets via ground-truth labels:


(9) $$S_{\text{pos}}=\left\{p(y=1|x_{i})|y_{i}=1\right\},\;S_{\text{neg}}=\left\{p(y=1|x_{j})|y_{j}=0\right\},$$

where $S_{\text{pos}}$ contains the sensitive-class scores of true sensitive cells and $S_{\text{neg}}$ contains those of true resistant cells. As illustrated in Fig. 1c, the overlap penalty is defined as


(10) $$L_{\text{olp}}=\frac{1}{\left|S_{\text{pos}}||S_{\text{neg}}\right|}\sum_{s_{a}\in S_{\text{pos}}}\sum_{s_{u}\in S_{\text{neg}}}\text{max}\left(0,\mu +s_{u}-s_{a}\right).$$

where μ = 0.3 is the preset margin threshold. The final source classification loss combines cross-entropy and overlap penalty with weights:


(11) $$L_{\text{cls}}=\lambda_{c}L_{\text{ce}}+\lambda_{o}L_{\text{olp}}.$$

where $\lambda_{c},\lambda_{o}$ are fixed hyperparameters ($\lambda_{c}=1.0,\lambda_{o}=1.0$) that balance classification accuracy and inter-class separation to form wide-margin decision boundaries.

### 2.4 Total objective function

The unified training loss jointly optimizes source classification, cross-domain alignment, and target clustering consistency:


(12) $$L_{\text{total}}=L_{\text{cls}}+\lambda_{d}L_{\text{dom}}+\lambda_{e}L_{\text{ent}}.$$

The entropy weight $\lambda_{e}$ increases linearly to avoid premature confidence in target predictions, whereas the domain-alignment weight $\lambda_{d}$ follows a dynamic warm-up schedule. This allows the model to prioritize predictive feature learning during the early training stage and gradually strengthen domain alignment in later epochs. Detailed mathematical formulations of these schedules are provided in Section G.4, available as supplementary data at *Bioinformatics* online. After convergence, the auxiliary training modules are discarded, and predictions are generated using only the shared encoder and classifier:


(13) $$\hat{Y}=\mathrm{softmax}\left[C\left(E_{\text{sh}}(E,G)\right)\right].$$

## 3 Results

### 3.1 Datasets

To comprehensively evaluate the robustness and generalizability of CSGDA across diverse biological heterogeneities, five representative single-cell RNA-sequencing (scRNA-seq) datasets were utilized in this study. These datasets encompass a variety of challenging cross-domain scenarios, including cross-platform, cross-cell line, cross-tissue (monotherapy and combination therapy), and cross-metastasis adaptation. The source and target domain characteristics of the five experiments are summarized in Table 1.

### 3.2 Analysis of model performance

To distinguish the performance gains of the transfer paradigm from those of CSGDA’s specific architecture, we first evaluated CSGDA and six representative baseline models under the conventional Bulk-to-SC setting, as shown in Fig. 2 and Table 8, available as supplementary data at *Bioinformatics* online. We then compared the same CSGDA architecture under Bulk-to-SC and SC-to-SC settings while keeping the target dataset and training protocol fixed, as reported in Table 10, available as supplementary data at *Bioinformatics* online. Finally, the comparison under the common SC-to-SC setting in Table 2, together with the module-level ablations in Fig. 3, was used to evaluate the architectural contribution of CSGDA.

**Table 1:** Summary of the five SC-to-SC domain adaptation experiments.^a^

Exp.	Drug	Scenario	Domain	Dataset	Platform	Biological origin	Cells (*S/R*).	Retained genes.
1	PLX4720	Cross-platform	Source	GSE108383	NextSeq	Melanoma, 451Lu	163 (80/83)	12 400
			Target	GSE108394	HiSeq	Melanoma, 451Lu	6548 (3286/3262)	14 341
2	PLX4720	Cross-cell line	Source	GSE108383	NextSeq	Melanoma, 451Lu	163 (80/83)	12 400
			Target	GSE108383	NextSeq	Melanoma, A375	101 (58/43)	9750
3	Paclitaxel	Cross-tissue (monotherapy).	Source	GSE169246	HiSeq X Ten.	PBMC	5183 (2022/3161)	7427
			Target	GSE169246	HiSeq X Ten.	Tumor tissue	2851 (765/2086)	9359
4	Paclitaxel + Atezolizumab.	Cross-tissue (combination).	Source	GSE169246	HiSeq X Ten.	PBMC	2053 (840/1213)	10 677
			Target	GSE169246	HiSeq X Ten.	Tumor tissue	4612 (1208/3404)	10 163
5	Cisplatin	Cross-metastasis	Source	GSE117872	HiSeq 2500.	Primary OSCC	611 (438/173)	22 744
			Target	GSE117872	HiSeq 2500.	Lymph-node metastatic OSCC.	429 (250/179)	22 744

a *S/R* denotes sensitive/resistant cells. Retained genes denotes the number of genes remaining in each domain after preprocessing.

**Table 2:** Performance comparison between CSGDA and baseline models on five datasets.

Method	Exp1: cross-platform				Exp2: cross-cell line				Exp3: cross-tissue (monotherapy)				Exp4: cross-tissue (combination)				Exp5: Cross-metastasis			
	ACC	F1-macro	AUC	AUPR	ACC	F1-macro	AUC	AUPR	ACC	F1-macro	AUC	AUPR	ACC	F1-macro	AUC	AUPR	ACC	F1-macro	AUC	AUPR
scDEAL	0.931	0.928	0.980	0.984	0.524	0.368	0.678	0.697	0.585	0.642	0.681	0.790	0.688	0.552	0.818	0.596	0.693	0.583	0.692	0.551
SCAD	0.992	0.992	0.998	0.998	0.871	**0.890**	0.882	0.914	0.831	0.811	0.911	0.859	0.834	0.817	0.899	0.824	0.794	0.798	0.849	0.885
scADADrug	0.958	0.962	**1.000**	**1.000**	0.541	0.666	0.696	0.850	0.839	0.785	0.906	0.857	0.863	0.846	0.898	0.767	0.554	0.713	0.399	0.497
scGSDR	0.984	0.984	0.999	0.999	0.673	0.672	0.793	0.834	0.674	0.611	0.720	0.597	0.576	0.473	0.533	0.419	0.564	0.412	0.615	0.701
SSDA4Drug	0.930	0.929	0.998	0.998	0.831	0.826	0.894	0.873	0.851	0.843	0.913	0.847	0.814	0.802	0.912	0.842	0.782	0.774	0.850	0.899
**CSGDA**	**0.994**	**0.994**	0.999	0.999	**0.891**	**0.890**	**0.931**	**0.952**	**0.857**	**0.846**	**0.924**	**0.885**	**0.912**	**0.907**	**0.970**	**0.964**	**0.882**	**0.873**	**0.966**	**0.957**

Bold values indicate the best performance for each metric in each experiment.

To address the inherent class imbalance in scRNA-seq data, we evaluated CSGDA using AUROC, AUPR, ACC, and F1-macro, systematically comparing it against five state-of-the-art methods across five scenarios (Table 2). To assess run-to-run variability, CSGDA, SCAD, and SSDA4Drug were further evaluated over 10 independent runs. Mean ± SD results are reported in Table 6, available as supplementary data at *Bioinformatics* online, and paired two-sided Wilcoxon signed-rank tests on matched random seeds are presented in Figs 2 and 3, available as supplementary data at *Bioinformatics* online. Runtime and peak GPU memory across the five tasks are reported in Table 11, available as supplementary data at *Bioinformatics* online. All methods received the same source–target pairs, matched gene-expression features, preprocessing procedures, data splits, and evaluation metrics, while retaining their model-specific feature transformations and training procedures. Official implementations and recommended or default settings were used for the baselines without target-specific tuning, whereas CSGDA parameters were selected using source-domain validation and fixed across all five tasks. CSGDA achieved robust performance overall, particularly in the cross-platform scenario, where it attained near-perfect scores alongside scAdaDrug and scGSDR; UMAP visualization (Fig. 4a and b, left panel) attributes this to the high intrinsic separability of resistance and sensitivity classes in low-noise settings.

**Figure 2:**
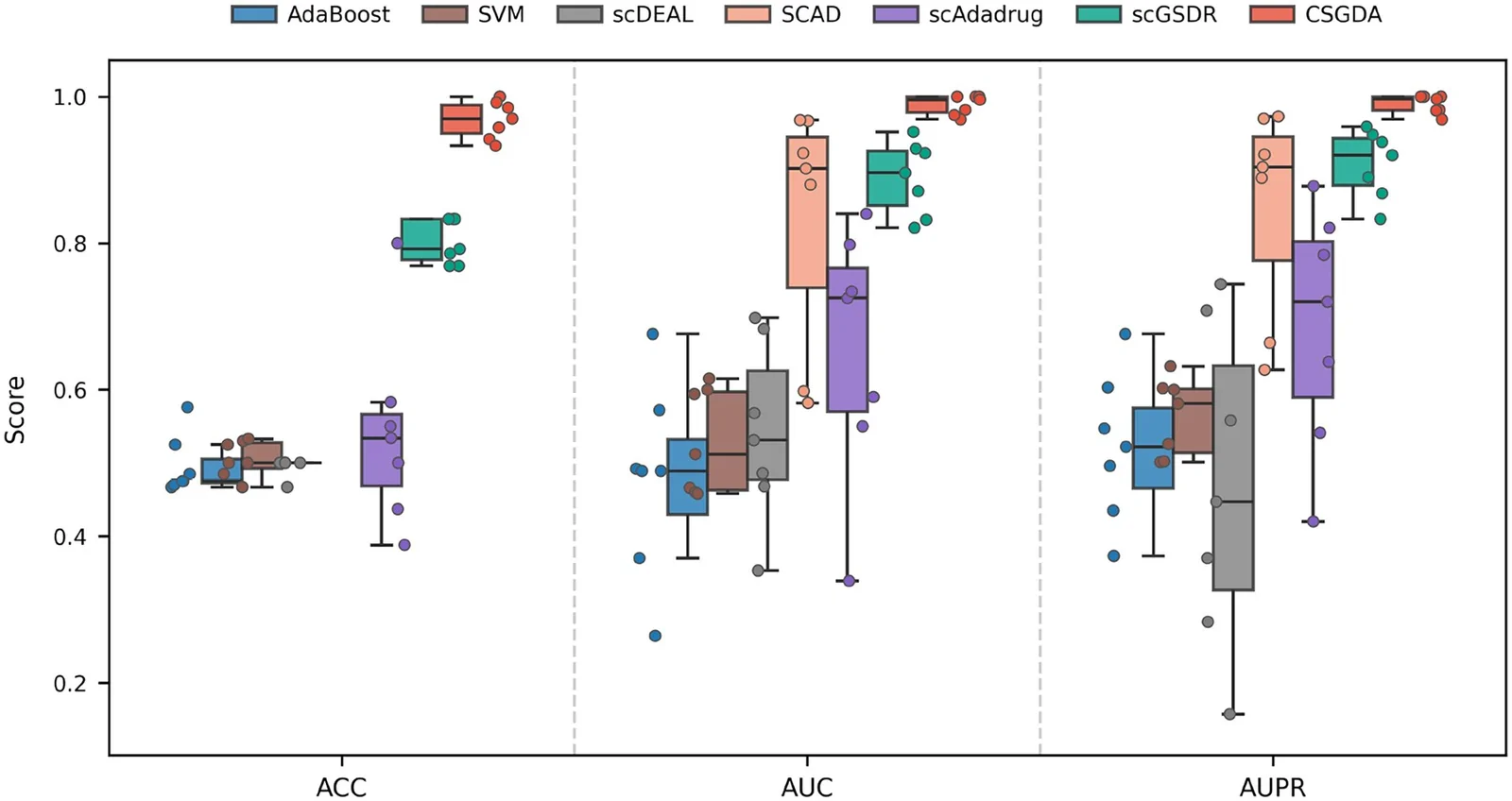
Overall performance distribution of CSGDA and six baseline models under the traditional bulk-to-SC transfer paradigm.

**Figure 3:**
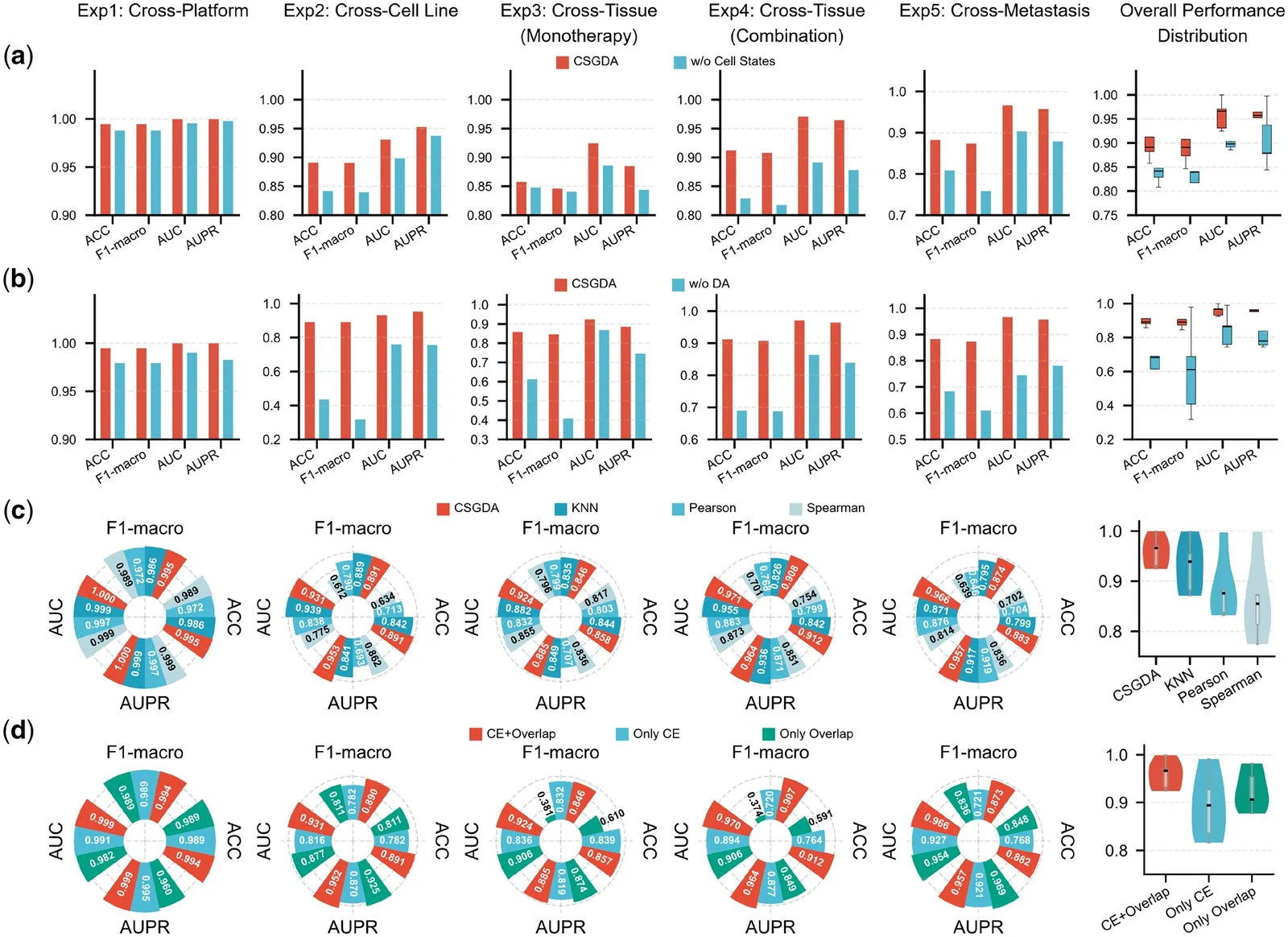
Comprehensive ablation study of CSGDA components across five experimental scenarios. (a) Impact of biological priors. (b) Contribution of the domain adaptation module. (c) Performance comparison of the CSA-GSL module against static graph construction strategies. (d) Efficacy of the hybrid loss function strategy.

However, as the scenarios progressed towards higher heterogeneity, performance stratification emerged, revealing the limitations of baseline architectures. First, unlike scDEAL which relies on statistical alignment (e.g. MMD), CSGDA employs a cell state-guided strategy. By aligning domains within a biological functional space rather than a purely statistical one, CSGDA better maintains performance when facing complex biological shifts. Second, while SCAD and scAdaDrug process cells as independent instances, CSGDA utilizes Dynamic Structure Learning. This allows it to capture non-linear topological relationships and intercellular dependencies, which are critical for identifying resistance in highly heterogeneous environments. Among the baselines, SSDA4Drug exhibited the strongest competitiveness. CSGDA, SCAD, scDEAL, scAdaDrug, and scGSDR used no target response labels during training, validation, hyperparameter tuning, or model selection, and target labels were accessed only for final evaluation. SSDA4Drug was reported separately as a semi-supervised comparator because it uses partial target labels during training. Despite this “information disadvantage,” CSGDA surpassed SSDA4Drug in the complex scenarios.

UMAP visualization reveals the discriminative power of CSGDA’s latent embeddings (compare Fig. 3a and b). In the Cross-Platform task, CSGDA effectively preserved clear class boundaries and compressed intra-class distances (Fig. 4b, left panel), confirming its robustness in baseline tasks. Crucially, in the high-noise Cross-Tissue(Monotherapy) scenario, the model transformed entangled raw data into a distinct “Inverted U-shape Manifold” (Fig. 4a and b, middle panel). This topology implies a continuous biological gradient rather than discrete clustering, suggesting that CSGDA captures the phenotypic plasticity and intermediate states traversing from sensitivity to resistance. Similarly, in the complex Cross-Tissue(Combination) setting, the model resolved irregular distributions into a compact “Hook-like Topology” (Fig. 4b, right panel), demonstrating strong non-linear feature extraction guided by cell states. Sankey diagrams (Fig. 4c) further quantify this alignment, showing high consistency between predictions and ground truth, particularly for the challenging samples in these cross-tissue scenarios.

### 3.3 Ablation study

To validate the contribution of each component in CSGDA, we conducted systematic ablation studies across five experimental scenarios. We compared the full model against variants lacking biological priors, dynamic graph learning, domain adaptation, and hybrid loss functions, respectively. As illustrated in the comprehensive performance distributions (Fig. 3), CSGDA demonstrated superior performance across most experimental scenarios and metrics, validating the synergy of the proposed modules. The corresponding ACC and AUPR values, together with the performance decreases relative to the full model, are reported in Table 5, available as supplementary data at *Bioinformatics* online.

**Figure 4:**
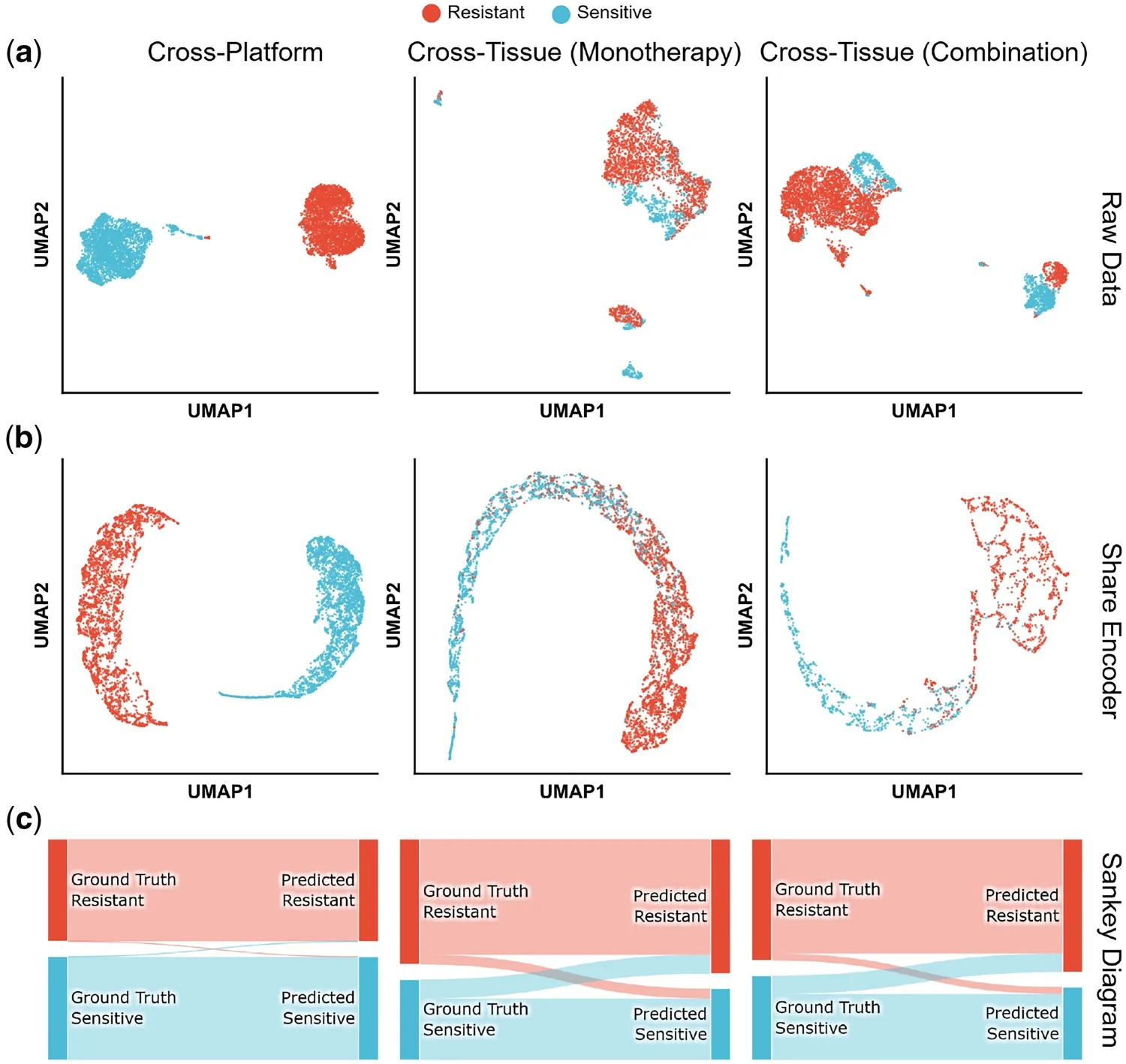
Feature alignment and prediction visualization. (a, b) UMAP comparison of raw data (a) and shared encoder (b) features across cross-platform, monotherapy, and combination tasks. (c) Sankey diagrams showing prediction consistency.

**Figure 5:**
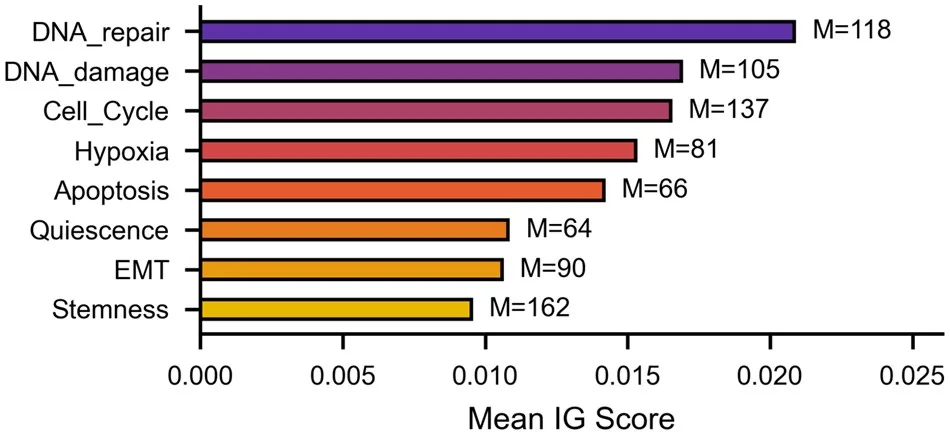
Global importance ranking of functional states. The bar chart displays the mean IG scores of high-contribution genes across eight predefined CancerSEA states.

#### 3.3.1 Impact of biological priors

We first verified the superiority of introducing cell states over purely data-driven features. Compared to the variant using PCA and K-Means (w/o Cell States), CSGDA exhibits significant robustness in complex environments (Fig. 3a). For instance, in the highly heterogeneous cross-tissue scenario, CSGDA maintained a high AUC of ∼0.97, whereas the statistical feature variant dropped to 0.89. This disparity confirms that biological priors provide conservative feature anchors (e.g. DNA repair semantics), effectively suppressing the model from learning spurious correlations from stochastic scRNA-seq noise.

#### 3.3.2 Effectiveness of CSA-GSL

We then assessed the graph learning module against static strategies (KNN, Pearson, Spearman), as illustrated in (Fig. 3c). Static methods failed to capture complex dependencies in the cross-metastasis scenario, with AUC scores stagnating below 0.88. In contrast, CSA-GSL achieved a remarkable AUC of 0.966. This improvement stems from the shift from “static feature similarity” to “dynamic semantic consistency,” where the graph structure is iteratively reconfigured based on updating predictions, correcting erroneous connections caused by dropout events.

#### 3.3.3 Contribution of domain adaptation

The exclusion of the domain adversarial network (w/o DA) precipitated a drastic performance collapse in cross-domain tasks (Fig. 3b). Specifically, in the combination therapy scenario, the absence of DA caused the AUC and AUPR to regress sharply from 0.97 and 0.964 to 0.863 and 0.839, respectively. This gap (>0.1) demonstrates that domain alignment serves as a crucial bridge, mitigating inter-domain distribution discrepancies (e.g. tissue origins) to ensure reliability on unseen targets.

#### 3.3.4 Necessity of cross-entropy and overlap loss

Finally, Fig. 3d highlights the limitations of single-modal losses. Relying solely on overlap loss ($L_{\text{olp}}$) in Exp. 4 resulted in a random-level F1-macro (0.374) despite high-ranking metrics, due to the lack of probability calibration. Conversely, using only cross-entropy ($L_{\text{ce}}$) in the tumor evolution scenario (Exp. 5) led to overfitting source boundaries. By organically integrating absolute probability supervision ($L_{\text{ce}}$) with relative topological constraints ($L_{\text{olp}}$), CSGDA achieves peak F1-macro scores (e.g. 0.873 in Exp. 5), ensuring both decision boundary precision and generalization.

### 3.4 Biological interpretability analysis

To assess whether the CSGDA model captures genuine biological mechanisms underlying drug resistance, we employed the IG algorithm to establish a multi-level “Drug Response–Gene–State” validation framework. Here, we focused on the challenging cross-metastasis scenario predicting cisplatin response (Experiment 5) as our representative case study. Our core logic involved identifying the genes that contribute most to resistance or sensitivity predictions (based on IG Scores), examining the enrichment patterns of these key genes within biological cell states, and subsequently inferring the biological mechanisms driving the model’s decisions.

#### 3.4.1 Identification and validation of driver genes

We first analyzed the core gene expression patterns that drive the model to predict resistance. As shown in Table 12, available as supplementary data at *Bioinformatics* online, the top 20 high-contribution genes identified by the model exhibited substantial IG scores, suggesting that the model mathematically regards the high expression of these specific genes as a significant determinant of cellular drug resistance. This computational finding aligns closely with clinical observations. Notably, RAD51C was identified as the top-ranking driver. As a paralog of RAD51, RAD51C is an essential component of the homologous recombination repair pathway; it assists in the assembly of the RAD51 recombinase on single-stranded DNA and plays a crucial role in repairing cisplatin-induced double-strand breaks (Hoppe *et al.* 2021). Clinical studies have indicated that elevated RAD51C expression is closely associated with cisplatin resistance and poor prognosis in various cancers, including ovarian and oral squamous cell carcinomas (Galluzzi *et al.* 2012, Jiang *et al.* 2024). The model’s assignment of high weight to RAD51C suggests it is detecting genuine resistance drivers rather than relying on data noise.

#### 3.4.2 Cell state mapping and resistance mechanisms

We utilized the CancerSEA database as a source of biological prior knowledge to bridge the gap between “micro-level genes” and “macro-level cell states.” Specifically, we employed gene signature sets from eight cancer-related functional states (e.g. DNA Repair, Hypoxia) as a mapping dictionary. By matching the high-contribution genes identified by CSGDA with this dictionary and calculating the IG scores for genes within each state, we quantified the overall contribution of different cell states to the resistance prediction (Fig. 5). This mapping analysis revealed a distinct pattern: a majority of high-contribution genes (such as RAD51C and ERCC1) aligned with the DNA Repair and DNA Damage states. Consequently, at the macro-state level, the DNA repair mechanism exhibited a predominant contribution. This result is consistent with the pharmacological mechanism of cisplatin, which aims to eliminate cancer cells via DNA damage, prompting resistant cells to upregulate DNA repair pathways to survive.

**Figure 6:**
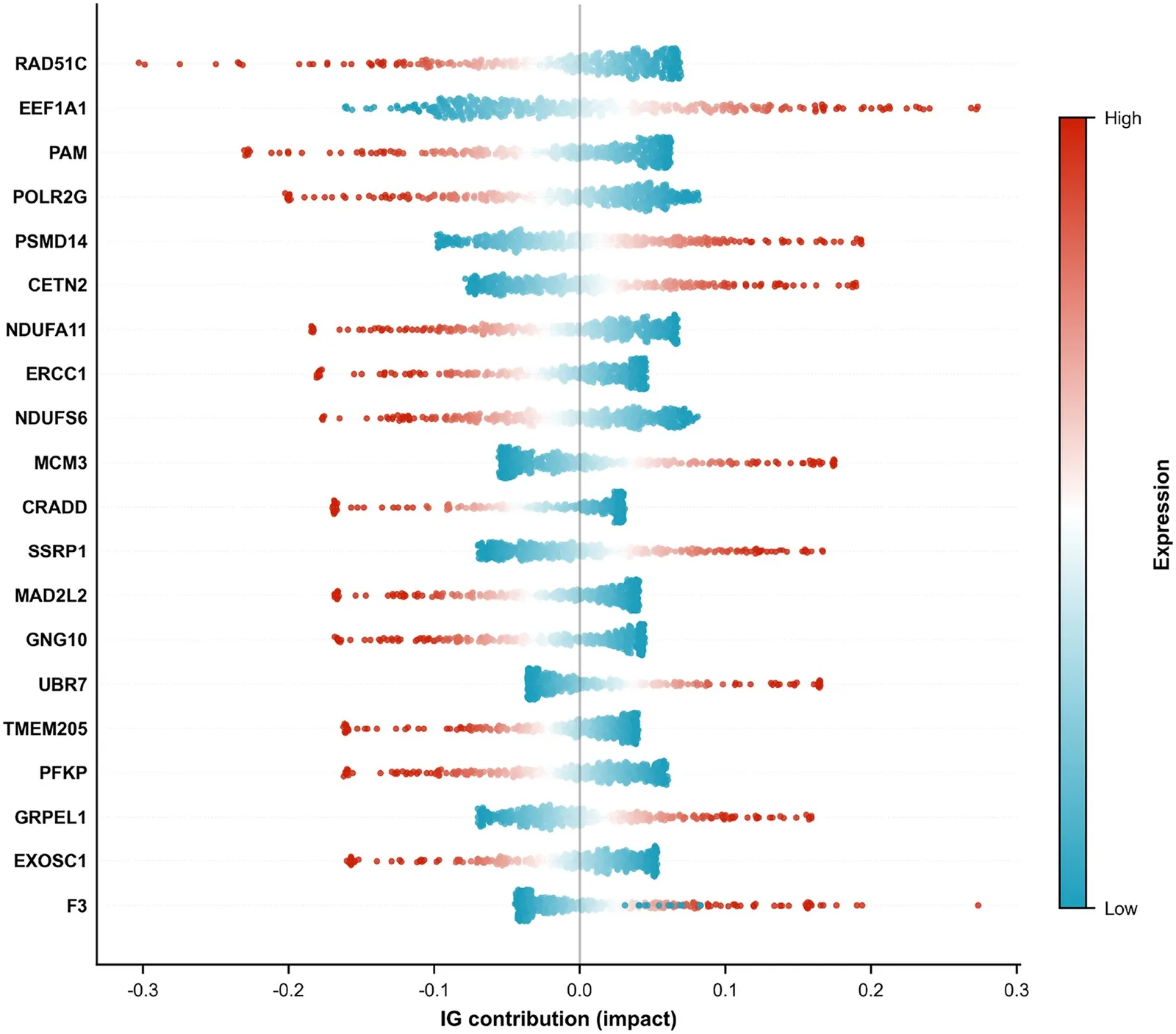
Beeswarm plot of top driver genes. The clustering of high-expression dots on the positive side demonstrates their strong driving force toward cisplatin resistance.

Additionally, the model identified Cell Cycle and Hypoxia as secondary factors. Cisplatin-induced damage typically triggers G2/M phase arrest, providing a window for DNA repair. Resistant cells often possess flexible cell cycle regulation, allowing them to evade apoptosis signals and rapidly resume proliferation (Sarin *et al.* 2017, Olszewska *et al.* 2021). Hypoxia within tumors can induce HIF-1α expression, which reshapes metabolic modes (e.g. by enhancing glycolysis) and transmits resistance signals to surrounding cells via mechanisms such as exosomes (Wang *et al.* 2021). The model’s detection of hypoxia reflects an understanding of complex resistance patterns driven by tumor microenvironmental stress. This indicates that CSGDA captures not only the primary drivers but also the auxiliary roles of tumor proliferation and microenvironmental pressure in drug resistance.

#### 3.4.3 Unsupervised discovery beyond priors

A key advantage of this framework is its ability to discover features beyond the boundaries of predefined knowledge. As shown in Fig. 6, the model identified a group of significant high-contribution genes—including NDUFA11, NDUFS6, and TMEM205—in an unsupervised manner. Although these genes were not included in the initial CancerSEA dictionary, the model detected their strong contribution to resistance based on intrinsic data distributions.

The identification of TMEM205, in particular, offers strong biological support for the model’s predictive mechanism. Literature confirms that TMEM205 is a 21 kDa transmembrane protein, primarily localized to the cell surface and the trans-Golgi network, and is specifically highly expressed in cisplatin-resistant cells (Shen *et al.* 2010). Its core resistance mechanism involves mediating the intracellular sequestration and active efflux of cisplatin, thereby reducing the effective drug dosage that reaches the nuclear DNA. The ability of CSGDA to independently identify this key efflux pump demonstrates that the framework can validate existing knowledge and has the potential to uncover implicit biological patterns, offering valuable leads for discovering novel resistance targets.

To assess the generalizability of these findings beyond cisplatin, we repeated the same IG analysis on the Paclitaxel cross-tissue dataset. The resulting gene rankings and biological interpretation are provided in Table 13, available as supplementary data at *Bioinformatics* online.

## 4 Conclusion and discussion

In this work, we present CSGDA, a cell state-guided graph domain adaptation framework for cross-domain single-cell drug response prediction. By leveraging CancerSEA functional priors to guide dynamic graph learning, CSGDA achieves superior and robust performance across diverse heterogeneous scenarios, and provides interpretable insights into drug resistance mechanisms. Future work will focus on improving scalability for large cell atlases and extending the framework to multi-omics integration.

## Supplementary Material

btag726_Supplementary_Data

## Data Availability

The CSGDA source code is available at https://github.com/yanfen-git/CSGDA-code and archived on Zenodo at https://doi.org/10.5281/zenodo.21759274. The datasets used in this study are available on Zenodo at https://doi.org/10.5281/zenodo.21770737. The sources and accession numbers of the original datasets are provided in the manuscript and Materials, available as supplementary data at *Bioinformatics* online.

## References

[btag726-B1] Chen J , Wang X, Ma A et al Deep transfer learning of cancer drug responses by integrating bulk and single-cell RNA-seq data. Nat Commun 2022;13:6494. 10.1038/s41467-022-34277-7 PMC9618578 36310235

[btag726-B2] Galluzzi L , Senovilla L, Vitale I et al Molecular mechanisms of cisplatin resistance. Oncogene 2012;31:1869–83. 10.1038/onc.2011.384 21892204

[btag726-B3] Ghorbian S. Cancer cell plasticity and therapeutic resistance: mechanisms, crosstalk, and translational perspectives. Hereditas 2025;162:188. 10.1186/s41065-025-00564-8 PMC12465352 41013839

[btag726-B4] Gundem G , Van Loo P, Kremeyer B et al The evolutionary history of lethal metastatic prostate cancer. Nature 2015;520:353–7. 10.1038/nature14347 PMC4413032 25830880

[btag726-B5] Hoppe MM , Jaynes P, Wardyn JD et al Quantitative imaging of RAD51 expression as a marker of platinum resistance in ovarian cancer. EMBO Mol Med 2021;13:e13366. 10.15252/emmm.202013366 PMC8103098 33709473

[btag726-B6] Huang D , Ma N, Li X et al Advances in single-cell RNA sequencing and its applications in cancer research. J Hematol Oncol 2023;16:98. 10.1186/s13045-023-01494-6 PMC10463514 37612741

[btag726-B7] Huang K , Liu H. Identification of drug-resistant individual cells within tumors by semi-supervised transfer learning from bulk to single-cell transcriptome. Commun Biol 2025;8:530. 10.1038/s42003-025-07959-3 PMC11958800 40164749

[btag726-B8] Huang Y-A , Cao X, You Z-H et al scGSDR: harnessing gene semantics for single-cell pharmacological profiling. Commun Biol 2025;8:1508. 10.1038/s42003-025-08788-0 PMC12572245 41162674

[btag726-B9] Hui HWH , Kong W, Goh WWB. Thinking points for effective batch correction on biomedical data. Brief Bioinform 2024;25:bbae515. 10.1093/bib/bbae515 PMC11471903 39397427

[btag726-B10] Jiang C , Shen C, Ni M et al Molecular mechanisms of cisplatin resistance in ovarian cancer. Genes Dis 2024;11:101063. 10.1016/j.gendis.2023.06.032 PMC11367050 39224110

[btag726-B11] Jin W , Ma Y, Liu X et al Graph structure learning for robust graph neural networks. In: *Proceedings of the 26th ACM SIGKDD International Conference on Knowledge Discovery & Data Mining*, New York NY: Association for Computing Machinery, 2020, p. 66–74.

[btag726-B12] Liu H , Duan W, Luo J. Predicting single-cell drug sensitivity utilizing adaptive weighted features for multi-source domain adaptation. IEEE J Biomed Health Inform 2025;29:6043–54. 10.1109/JBHI.2025.3553126 40111773

[btag726-B13] Maeser D , Zhang W, Huang Y et al A review of computational methods for predicting cancer drug response at the single-cell level through integration with bulk RNA-seq data. Curr Opin Struct Biol 2024;84:102745. 10.1016/j.sbi.2023.102745 PMC10922290 38109840

[btag726-B14] Marusyk A , Janiszewska M, Polyak K. Intratumor heterogeneity: the rosetta stone of therapy resistance. Cancer Cell 2020;37:471–84. 10.1016/j.ccell.2020.03.007 PMC7181408 32289271

[btag726-B15] Olszewska A , Borkowska A, Granica M et al Escape from cisplatin-induced senescence of hypoxic lung cancer cells can be overcome by hydroxychloroquine. Front Oncol 2021;11:738385. 10.3389/fonc.2021.738385 PMC8813758 35127467

[btag726-B16] Park Y , Hauschild A-C, Heider D. Transfer learning compensates limited data, batch effects and technological heterogeneity in single-cell sequencing. NAR Genom Bioinform 2021;3:lqab104. 10.1093/nargab/lqab104 PMC8598306 34805988

[btag726-B17] Sarin N , Engel F, Kalayda GV et al Cisplatin resistance in non-small cell lung cancer cells is associated with an abrogation of cisplatin-induced G2/M cell cycle arrest. PLoS One 2017;12:e0181081. 10.1371/journal.pone.0181081 PMC5528889 28746345

[btag726-B18] Savino A , Iannuzzi RM, Avalle L et al Cross-tissue gene expression interactions from bulk, single cell and spatial transcriptomics with crosswgcna. BMC Genomics 2025;26:583. 10.1186/s12864-025-11747-y PMC12211745 40597567

[btag726-B19] Shen D-W , Ma J, Okabe M et al Elevated expression of TMEM205, a hypothetical membrane protein, is associated with cisplatin resistance. J Cell Physiol 2010;225:822–8. 10.1002/jcp.22287 PMC2971691 20589834

[btag726-B20] Van de Sande B , Lee JS, Mutasa-Gottgens E et al Applications of single-cell RNA sequencing in drug discovery and development. Nat Rev Drug Discov 2023;22:496–520. 10.1038/s41573-023-00688-4 PMC10141847 37117846

[btag726-B21] Wang D , Zhao C, Xu F et al Cisplatin-resistant NSCLC cells induced by hypoxia transmit resistance to sensitive cells through exosomal PKM2. Theranostics 2021;11:2860–75. 10.7150/thno.51797 PMC7806469 33456577

[btag726-B22] Yan F , Du Z, Huang Y-A. DAGFormer: a graph-based domain adaptation approach for single-cell cancer drug response prediction. PLoS Comput Biol 2025;21:e1013832. 10.1371/journal.pcbi.1013832 PMC12795466 41417875

[btag726-B23] Yuan H , Yan M, Zhang G et al Cancersea: a cancer single-cell state atlas. Nucleic Acids Res 2019;47:D900–8. 10.1093/nar/gky939 PMC6324047 30329142

[btag726-B24] Zheng Z , Chen J, Chen X et al Enabling single-cell drug response annotations from bulk RNA-Seq using SCAD. Advanced Science 2023;10:2204113. 10.1002/advs.202204113 PMC10104628 36762572

