# Supplementary material for: CSGDA: a cell state-guided graph domain adaptation network for single-cell drug response prediction
Source: Bioinformatics. 2026 Sep 29;42(10):btag726. doi: 10.1093/bioinformatics/btag726 (PMC13680098; doi:10.1093/bioinformatics/btag726)
Supplement: Supplementary file 1 [file btag726_supplementary_data.pdf]

Appendix A Dataset Description

To comprehensively evaluate the robustness of the CSGDA model in handling inherent distribution shifts and biological heterogeneity within single-cell transcriptomic data, we collected and curated five representative scRNA-seq datasets from the DRMref and NCBI GEO databases. These datasets are meticulously designed to form four progressive domain adaptation scenarios, ranging from technical batch effects introduced by sequencing platforms to complex evolutionary mechanisms of tumor metastasis. Detailed dataset information is summarized in Table S1, Table S2, and Fig. S1.

Table S1: Summary of Source Domain Datasets

Exp. ID	Drug/Regimen	Adaptation Scenario	Sensitive	Resistant	GEO Accession
Exp. 1	PLX4720	Cross-Platform	80	83	GSE108383
Exp. 2	PLX4720	Cross-Cell Line	80	83	GSE108383
Exp. 3	Paclitaxel	Cross-Tissue	2,022	3,161	GSE169246
Exp. 4	Paclitaxel & Atezolizumab	Cross-Tissue & Combination	840	1,213	GSE169246
Exp. 5	Cisplatin	Cross-Metastasis	438	173	GSE117872

Table S2: Summary of Target Domain Datasets

Exp. ID	Drug/Regimen	Adaptation Scenario	Sensitive	Resistant	GEO Accession
Exp. 1	PLX4720	Cross-Platform	3,286	3,262	GSE108394
Exp. 2	PLX4720	Cross-Cell Line	58	43	GSE108383
Exp. 3	Paclitaxel	Cross-Tissue	765	2,086	GSE169246
Exp. 4	Paclitaxel & Atezolizumab	Cross-Tissue & Combination	1,208	3,404	GSE169246
Exp. 5	Cisplatin	Cross-Metastasis	250	179	GSE117872

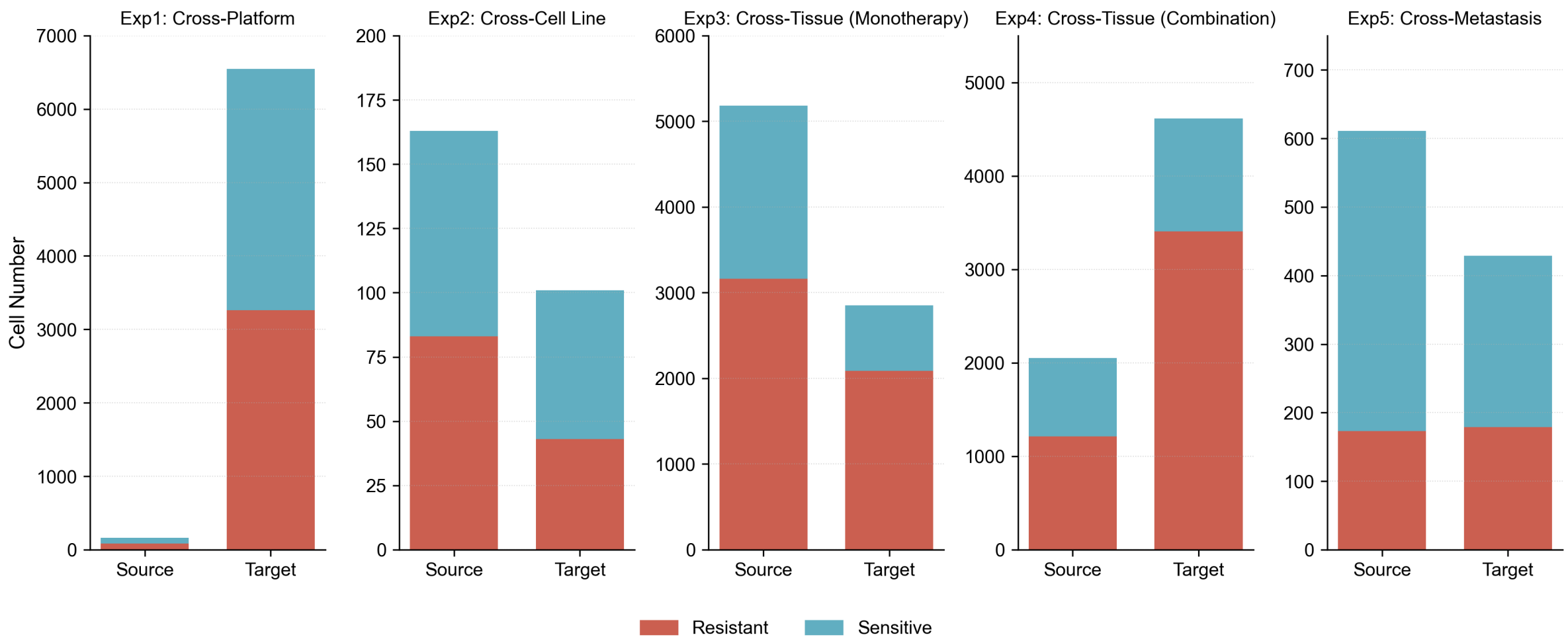

Figure S1: Distribution statistics of resistant and sensitive cells in source and target domains across five experimental scenarios.

Experiment 1: Cross-Platform Adaptation Verification. To evaluate the model's ca-

pability to overcome systemic noise introduced by different sequencing technologies, we designed a cross-platform experiment for the drug PLX4720 [10]. The source domain data originates from GSE108383, generated by the NextSeq platform, containing 163 melanoma cells (83 resistant, 80 sensitive); the target domain data originates from GSE108394, generated by the HiSeq platform, containing 6,548 cells (3,262 resistant, 3,286 sensitive). This experiment aims to verify whether the model, trained on small-scale, low-depth sequencing data, can effectively generalize to large-scale, high-depth sequencing data, thereby resolving cross-platform batch effects.

Experiment 2: Cross-Cell Line Heterogeneity Generalization. To verify the model's transferability across different genetic backgrounds, we constructed a cross-cell line experiment for PLX4720. Data were sourced from the GSE108383 dataset. We used the 451Lu melanoma cell line (163 cells: 83 resistant, 80 sensitive) as the source domain and the A375 cell line (101 cells: 43 resistant, 58 sensitive) as the target domain. This experiment aims to simulate whether resistance patterns learned from a cell line with a clear genetic background can be accurately transferred to another cell line with distinct genetic characteristics.

Experiments 3 & 4: Cross-Tissue Microenvironment Transfer. Considering the significant discrepancies between the tumor microenvironment and the peripheral blood environment, we utilized the GSE169246 [27] dataset to design two sets of cross-tissue experiments. These evaluate the feasibility of transferring knowledge from easily accessible peripheral blood mononuclear cells (PBMC) to solid tumor tissues. Overall, this addresses cross-tissue distribution shifts and verifies the potential of "liquid biopsy"-type data in assisting solid tumor drug response prediction. Specifically:

- **Experiment 3 (Single-drug scenario):** Focuses on Paclitaxel monotherapy. The source domain contains 5,183 PBMC cells, while the target domain contains 2,851 tumor tissue cells. This experiment aims to test the model's performance across tissue types in monotherapy settings.
- **Experiment 4 (Combination therapy scenario):** Focuses on the combination treatment of Paclitaxel and Atezolizumab. The source domain consists of 2,053 PBMC cells, and the target domain consists of 4,612 tumor tissue cells. This experiment aims to evaluate the model's efficacy in multi-drug combination scenarios, utilizing patient PBMC data to predict drug responses in tumor tissue cells.

Experiment 5: Cross-Metastasis Status Evolutionary Analysis. To explore the model's generalization ability during malignant tumor progression—specifically, whether it can leverage knowledge from primary tumors to predict more heterogeneous metastatic tumors—we designed a cross-metastasis experiment for Cisplatin. Data were sourced from GSE117872 (Sharma et al.), focusing on Oral Squamous Cell Carcinoma (OSCC). We used primary tumor cells as the source domain (611 cells: 173 resistant, 438 sensitive) and paired lymph node metastatic tumor cells as the target domain (429 cells: 179 resistant, 250 sensitive). This experiment focuses on examining whether the model can capture conserved resistance features across tumor evolutionary stages, addressing biological distribution shifts driven by metastasis, and verifying its clinical potential in handling tumor evolution and heterogeneity.

Traditional Bulk-to-SC Datasets.
Tables S3 and S4 summarize the bulk source and single cell target datasets used in the Bulk-to-SC experiments, including their class distributions and numbers of shared genes.

Appendix B Data Preprocessing

Bulk data preprocessing. The bulk expression profiles were obtained from GDSC in RMA normalized form. Duplicate gene identifiers were removed, and only genes shared with the

Table S3: **Source domain datasets under the traditional Bulk-to-SC paradigm.**

Drug	Cell line	Dataset	Resistant	Sensitive	R:S ratio	Genes
Gefitinib	Pan-Can	GDSC	714 (86.1%)	115 (13.9%)	6.21:1	10,610
Afatinib	Pan-Can	GDSC	682 (82.0%)	150 (18.0%)	4.55:1	10,684
AR-42	Pan-Can	GDSC	811 (91.0%)	80 (9.0%)	10.14:1	10,610
Cetuximab	Pan-Can	GDSC	739 (85.8%)	122 (14.2%)	6.06:1	10,684
Etoposide	Pan-Can	GDSC	811 (93.9%)	53 (6.1%)	15.30:1	9,738
NVP-TAE684	Pan-Can	GDSC	358 (90.6%)	37 (9.4%)	9.68:1	10,684
PLX4720	Pan-Can	GDSC	746 (54.3%)	629 (45.7%)	1.19:1	11,937
Sorafenib	Pan-Can	GDSC	362 (85.8%)	60 (14.2%)	6.03:1	10,684
Vorinostat	Pan-Can	GDSC	774 (92.8%)	60 (7.2%)	12.90:1	10,610

Note: Percentages were calculated within each drug-specific source dataset. Shared genes denotes the number of genes retained in both the bulk source and single-cell target domains.

Table S4: **Target domain datasets under the traditional Bulk-to-SC paradigm.**

Drug	Cell line	Dataset	Platform	Resistant	Sensitive	R:S ratio	Genes
Gefitinib	JUH006	CCLE	10x Genomics	33 (50.0%)	33 (50.0%)	1.00:1	10,610
Afatinib	SCC47	CCLE	10x Genomics	60 (50.0%)	60 (50.0%)	1.00:1	10,684
AR-42	JUH006	CCLE	10x Genomics	33 (50.0%)	33 (50.0%)	1.00:1	10,610
Cetuximab	SCC47	CCLE	10x Genomics	60 (50.0%)	60 (50.0%)	1.00:1	10,684
Etoposide	PC9	GSE149215	10x Genomics	764 (54.8%)	629 (45.2%)	1.21:1	9,738
NVP-TAE684	SCC47	CCLE	10x Genomics	60 (50.0%)	60 (50.0%)	1.00:1	10,684
PLX4720	451Lu	GSE108383	SMART-seq	63 (50.0%)	63 (50.0%)	1.00:1	11,937
PLX4720	A375	GSE108383	SMART-seq	48 (43.6%)	62 (56.4%)	0.77:1	11,937
Sorafenib	SCC47	CCLE	10x Genomics	60 (50.0%)	60 (50.0%)	1.00:1	10,684
Vorinostat	JUH006	CCLE	10x Genomics	33 (50.0%)	33 (50.0%)	1.00:1	10,610

Note: Percentages were calculated within each drug-specific target dataset. Shared genes denotes the number of genes retained in both the bulk source and single-cell target domains.

corresponding single cell target dataset were retained. The bulk source and single cell target matrices were then standardized independently using gene wise Z score transformation. Drug-response labels were derived from IC50 measurements using the LOBICO binary classification procedure. All available labeled bulk samples were used as the source training set.

Single cell data. All scRNA-seq data analyzed in this study were obtained from the Gene Expression Omnibus (GEO) of the National Center for Biotechnology Information (NCBI) [https://www.ncbi.nlm.nih.gov/geo/]. All datasets are accessible via the GEO Series accession numbers listed in Tables S1 and S2. Experiments 1-4 referenced data from DRMref [19]. Data preprocessing was conducted using the Scanpy pipeline, comprising two stages: Quality Control (QC) and Normalization.

Quality Control Stage: We implemented differentiated filtering strategies based on the sequencing depth and sample characteristics of each dataset:

- **Experiments 1 & 2**: For source domain data, we applied strict filtering criteria, retaining cells with detected gene counts $N_{genes} > 8,500$ (and $< 200,000$) and total molecular counts $> 10,000$ (and $< 2.7 \times 10^7$). For the target domain of Experiment 1, cells with $N_{genes} > 300$ and mitochondrial gene percentage $Pct_{mito} < 15\%$ were retained. Conversely, for the target domain of Experiment 2, which features higher sequencing depth, we retained cells with $N_{genes} > 7,000$ and total counts $> 1 \times 10^6$.

- **Experiments 3 & 4**: To accommodate the heterogeneity of cross-tissue data, mitochondrial percentage thresholds for both source and target domains were relaxed to $Pct_{mito} < 60\%$. The gene count threshold for Experiment 3 was set to $N_{genes} > 1,600$.

For Experiment 4, source and target domain thresholds were set to $N_{genes} > 1,300$ and $N_{genes} > 1,200$, respectively. Additionally, genes expressed in fewer than 50–100 cells were excluded, depending on the specific sparsity of the dataset.

- **Experiment 5**: We filtered out low-quality droplets (i.e., $N_{genes} < 200$) and cells with high mitochondrial content ($Pct_{mito} > 10\%$). Genes expressed in fewer than 3 cells were also excluded.

Normalization Stage: Post-QC data were first normalized to 1×10^5 total counts per cell, followed by $\log 1p$ transformation and Z-score standardization to construct the feature matrix for subsequent analysis.

Appendix C Cell state feature extraction and pseudo-label construction

This selection targets four pivotal biological mechanisms driving drug resistance:

- **Proliferation and Survival.** We incorporated **Cell Cycle**, **Apoptosis**, and **Quiescence**. Mechanisms such as cell cycle dysregulation, activation of anti-apoptotic pathways, and treatment evasion via entry into the G_0 resting phase represent primary strategies for circumventing cytotoxic therapy [16, 17, 25].

- **Genomic Integrity.** We included **DNA Damage** and **DNA Repair**. Enhanced DNA repair capacity serves as a critical defense mechanism against DNA-damaging agents, particularly platinum-based chemotherapies [15, 21, 26].

- **Cell Plasticity and Stemness.** We selected **Stemness** and **Epithelial-Mesenchymal Transition (EMT)**. These states collectively drive the emergence of aggressive cell subpopulations characterized by self-renewal capabilities and intrinsic multidrug resistance [8, 22, 24].

- **Microenvironmental Adaptation.** We selected **Hypoxia**. Hypoxic conditions within the tumor microenvironment activate pro-survival signaling (e.g., the HIF-1α pathway), shielding cells from drug-induced cytotoxicity [4, 23].

Together, the eight states define a complementary feature space covering major mechanisms of drug response and resistance. They were selected before model training, fixed across all source and target pairs, and not adjusted using target response labels or prediction performance. Missing marker genes were excluded, and a state score was set to zero when no markers were available. The state with the highest score was assigned as the pseudo label.

Appendix D Summary of Baseline Model Architectures

To provide context for the comparative analysis, we briefly describe the architectures of the five baseline models used in this study:

- **scDEAL** [1]: A deep transfer learning framework that bridges bulk and single-cell data. It employs a Deep Neural Network (DNN) coupled with an autoencoder and utilizes statistical alignment (MMD) to harmonize feature distributions between domains.

- **SCAD** [28]: An adversarial domain adaptation model designed for single-cell drug response annotation. It uses a generator-discriminator architecture to create domain-invariant features, transferring drug sensitivity labels from pre-clinical cell lines to patient-derived cells.

- **scAdaDrug** [18]: A multi-source domain adaptation framework featuring an adaptive weighting mechanism. It employs an importance-aware module to selectively weight different source datasets, optimizing the leverage of relevant information from multiple bulk references.
- **SSDA4Drug** [12]: A semi-supervised transfer learning method tailored for few-shot scenarios. It combines adversarial adaptation with consistency regularization techniques (e.g., MixMatch) to enhance performance when labeled single-cell data is scarce.
- **scGSDR** [14]: A biologically interpretable model that integrates gene semantics and pathway knowledge. It utilizes attention mechanisms to weigh specific signaling pathways, aiming to enhance the biological relevance of drug response predictions.

Ablation Analysis. To quantify the contributions of cell-state guidance, adaptive graph refinement, adversarial domain alignment, and the overlap penalty, we compared the full CSGDA model with four controlled variants. The *w/o Cell States* variant removes cell-state guidance, and the *Static KNN Graph* variant replaces the CSA-GSL-refined graph with the initial KNN graph. The *w/o Domain Adaptation* variant removes the domain discriminator, the GRL pathway, and $\mathcal{L}_{\text{dom}}$, whereas the *Only CE* variant removes $\mathcal{L}_{\text{olp}}$. All other model components and training settings were retained. The corresponding results are summarized in Table S5.

Table S5: Numerical summary of ablation results across five SC-to-SC tasks

Dataset	Variant	ACC	ΔACC	AUPR	ΔAUPR
PLX4720	Full CSGDA	0.9947	0.0000	0.9999	0.0000
	w/o Cell States	0.9878	+0.0069	0.9977	+0.0022
	w/o Domain Adaptation	0.9795	+0.0152	0.9826	+0.0173
	Static KNN Graph	0.9861	+0.0086	0.9991	+0.0008
	Only CE	0.9890	+0.0057	0.9950	+0.0049
PLX4720-A375	Full CSGDA	0.8911	0.0000	0.9527	0.0000
	w/o Cell States	0.8416	+0.0495	0.9375	+0.0152
	w/o Domain Adaptation	0.4356	+0.4555	0.7563	+0.1964
	Static KNN Graph	0.8416	+0.0495	0.8414	+0.1113
	Only CE	0.7820	+0.1091	0.8700	+0.0827
Paclitaxel	Full CSGDA	0.8576	0.0000	0.8850	0.0000
	w/o Cell States	0.8478	+0.0098	0.8439	+0.0411
	w/o Domain Adaptation	0.6132	+0.2444	0.7451	+0.1399
	Static KNN Graph	0.8441	+0.0135	0.8491	+0.0359
	Only CE	0.8390	+0.0186	0.8190	+0.0660
Paclitaxel–Atezolizumab	Full CSGDA	0.9123	0.0000	0.9643	0.0000
	w/o Cell States	0.8290	+0.0833	0.8780	+0.0863
	w/o Domain Adaptation	0.6897	+0.2226	0.8393	+0.1250
	Static KNN Graph	0.8422	+0.0701	0.9364	+0.0279
	Only CE	0.7640	+0.1483	0.8770	+0.0873
Cisplatin	Full CSGDA	0.8826	0.0000	0.9571	0.0000
	w/o Cell States	0.8082	+0.0744	0.8788	+0.0783
	w/o Domain Adaptation	0.6830	+0.1996	0.7807	+0.1764
	Static KNN Graph	0.7995	+0.0831	0.9168	+0.0403
	Only CE	0.7680	+0.1146	0.9210	+0.0361

Note: ΔACC and ΔAUPR were calculated as the performance of full CSGDA minus that of the corresponding variant. Positive values indicate performance reductions after removing or replacing the corresponding component.

Across all five tasks, removing cell-state guidance or replacing CSA-GSL with a static KNN graph reduced both ACC and AUPR. Removing domain adaptation produced the largest overall performance deterioration, while the Only CE variant also consistently underperformed the full model. These results support the complementary contributions of cell-state guidance, adaptive graph refinement, adversarial domain alignment, and overlap-aware classification.

Appendix E Supplementary Performance Stability Analysis

To evaluate the predictive stability beyond the mean values reported in Table 2, we first analyzed the comprehensive performance distributions across all experimental tasks using box plots (Figure S2). The results indicate that CSGDA not only achieves the highest median performance across all metrics but also exhibits a significantly more compact interquartile range (IQR). This demonstrates the model's superior robustness and predictive consistency when handling the inherent high noise and biological heterogeneity of single-cell transcriptomic data.

Subsequently, we conducted a detailed statistical significance analysis comparing CSGDA with representative baseline models (SCAD and SSDA4Drug) across the five experimental scenarios (Figure S3). Based on 10 independent runs with different random seeds, the paired Wilcoxon signed-rank test results confirm that the performance gains achieved by CSGDA in ACC, AUC, AUPR, and Macro F1-Score are statistically significant (* $p < 0.05$, ** $p < 0.01$, *** $p < 0.001$). This further validates the efficacy of the proposed cell state-guided and overlap-penalized domain adaptation framework in resolving challenging non-I.I.D. distribution shifts.

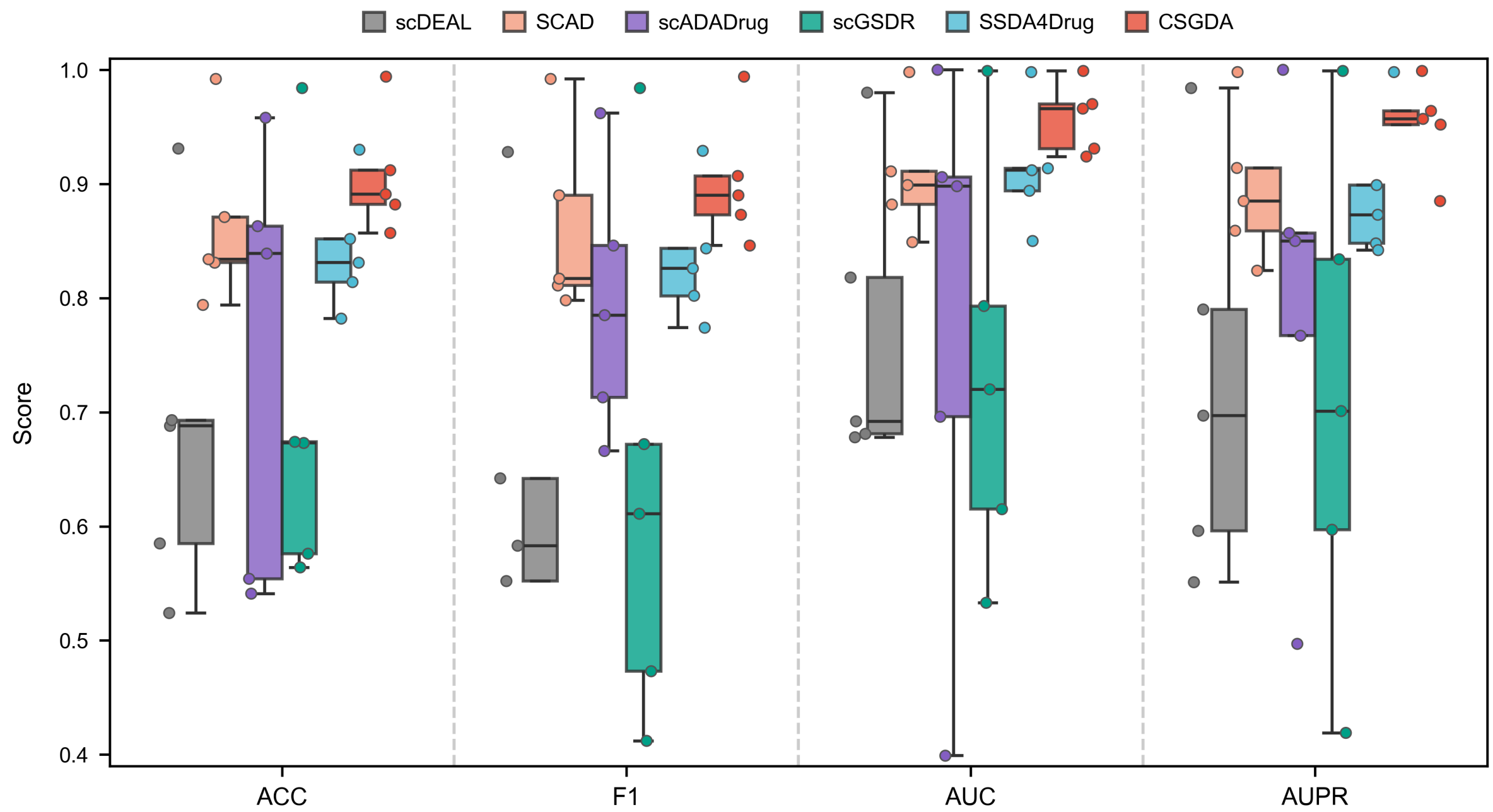

Figure S2: **Performance distribution benchmark.** Comparison of CSGDA against five baselines across all experimental tasks in terms of ACC, F1, AUC, and AUPR.

Repeated Run Robustness. To quantify run-to-run variability against strong comparators, we evaluated CSGDA together with SCAD, the strongest fully unsupervised baseline, and SSDA4Drug, the strongest semi-supervised comparator, over ten independent runs with different random seeds. The resulting mean values and standard deviations are summarized in Table S6.

Across all five tasks, CSGDA achieved the highest mean value for each metric among the three methods. Its standard deviations were no greater than 0.0365 for ACC, 0.0362 for macro-F1, 0.0281 for AUROC, and 0.0167 for AUPR. In the imbalanced Paclitaxel, Paclitaxel–Atezolizumab, and Cisplatin tasks, CSGDA maintained relatively small variability in macro-F1 and AUPR, indicating that its performance was not determined by a single favorable run.

Inductive Evaluation on Unseen Target Cells. To assess generalization beyond the target cells used for domain adaptation, we performed ten random splits of the target domain

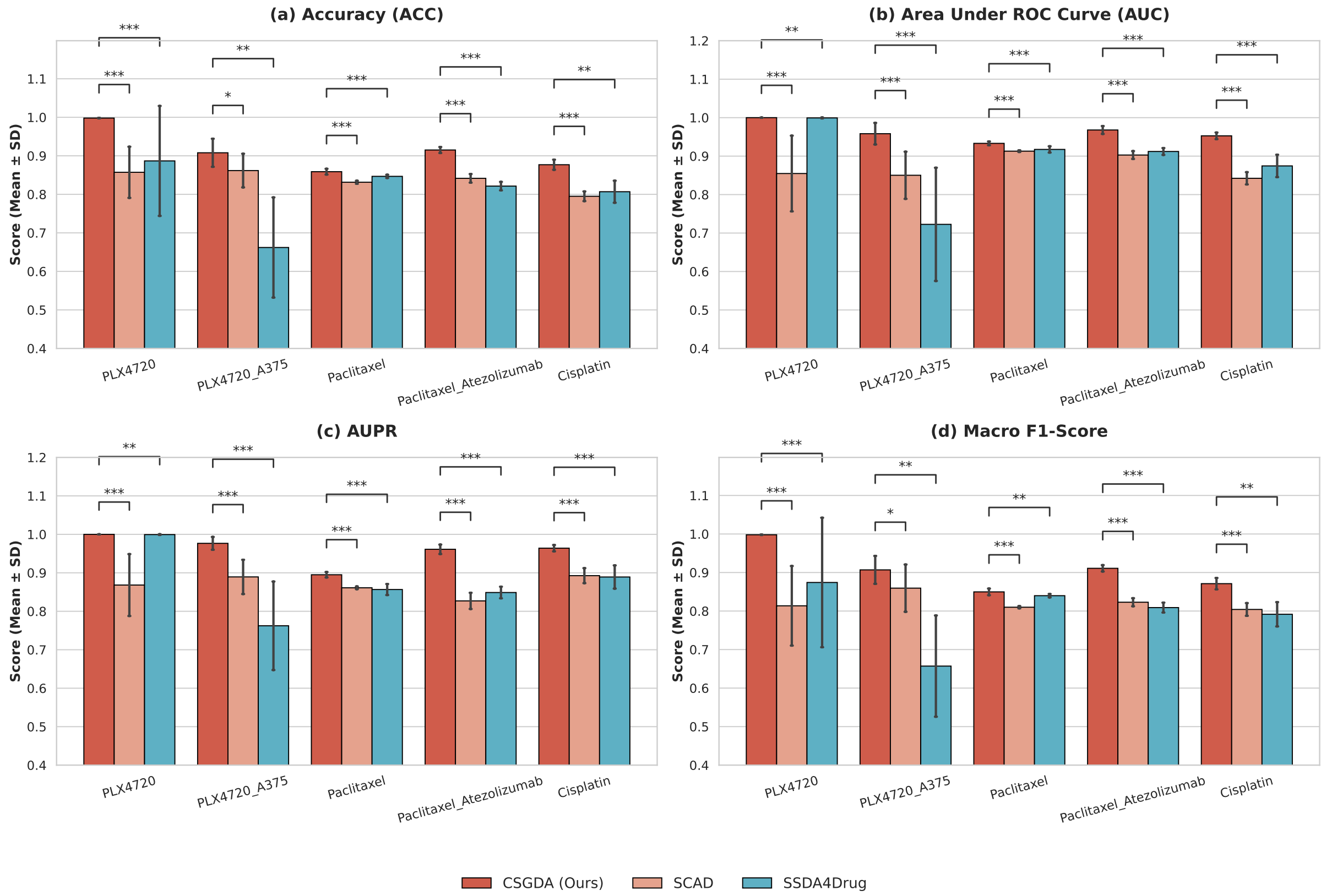

Figure S3: Performance comparison of CSGDA (Ours), SCAD, and SSDA4Drug across five datasets evaluated by (a) ACC, (b) AUC, (c) AUPR, and (d) Macro F1-Score. Bar heights represent the mean scores over 10 independent runs with different random seeds, and error bars indicate the standard deviations. Statistical significance between CSGDA and baselines was calculated using the paired Wilcoxon signed-rank test (* $p < 0.05$, ** $p < 0.01$, *** $p < 0.001$).

Table S6: Performance robustness across ten independent runs

Dataset	Method	ACC	Macro-F1	AUROC	AUPR
PLX4720	CSGDA	0.9980 ± 0.0006	0.9980 ± 0.0006	1.0000 ± 0.0000	1.0000 ± 0.0000
	SCAD	0.8572 ± 0.0664	0.8136 ± 0.1033	0.8548 ± 0.0984	0.8682 ± 0.0805
	SSDA4Drug	0.8866 ± 0.1420	0.8742 ± 0.1662	0.9993 ± 0.0011	0.9994 ± 0.0009
PLX4720-A375	CSGDA	0.9079 ± 0.0365	0.9069 ± 0.0362	0.9583 ± 0.0281	0.9766 ± 0.0167
	SCAD	0.8618 ± 0.0437	0.8595 ± 0.0614	0.8502 ± 0.0613	0.8893 ± 0.0445
	SSDA4Drug	0.6747 ± 0.1022	0.6682 ± 0.1048	0.7371 ± 0.1270	0.7733 ± 0.1011
Paclitaxel	CSGDA	0.8589 ± 0.0076	0.8498 ± 0.0087	0.9332 ± 0.0050	0.8950 ± 0.0071
	SCAD	0.8314 ± 0.0040	0.8099 ± 0.0029	0.9129 ± 0.0022	0.8611 ± 0.0036
	SSDA4Drug	0.8475 ± 0.0041	0.8395 ± 0.0045	0.9154 ± 0.0082	0.8549 ± 0.0152
Paclitaxel–Atezolizumab	CSGDA	0.9151 ± 0.0078	0.9110 ± 0.0083	0.9679 ± 0.0103	0.9612 ± 0.0123
	SCAD	0.8416 ± 0.0112	0.8229 ± 0.0105	0.9028 ± 0.0102	0.8270 ± 0.0211
	SSDA4Drug	0.8210 ± 0.0119	0.8089 ± 0.0135	0.9094 ± 0.0091	0.8449 ± 0.0170
Cisplatin	CSGDA	0.8769 ± 0.0131	0.8711 ± 0.0148	0.9528 ± 0.0086	0.9641 ± 0.0083
	SCAD	0.7950 ± 0.0126	0.8042 ± 0.0165	0.8424 ± 0.0160	0.8927 ± 0.0195
	SSDA4Drug	0.8121 ± 0.0277	0.8092 ± 0.0281	0.8853 ± 0.0247	0.9169 ± 0.0213

Note: Results are reported as the mean ± standard deviation over 10 independent runs with different random seeds.

into an 80% unlabeled adaptation subset and a 20% independent test subset without reference to response labels. Only the adaptation subset was used to estimate preprocessing parameters,

construct the target graph, and compute the domain-alignment and entropy-minimization losses. The test subset did not participate in preprocessing, training, or model selection. After training, all model parameters were fixed, a separate graph was constructed for the test subset, and performance was evaluated exclusively on these previously unseen cells.

Table S7: Inductive target-holdout performance of CSGDA over ten random splits. Results are reported as mean ± standard deviation.

Dataset	Adaptation/Test cells	ACC	Macro-F1	AUC	AUPR
PLX4720	5,238/1,310	0.9351 ± 0.1574	0.9202 ± 0.2044	0.9983 ± 0.0025	0.9980 ± 0.0029
PLX4720-A375	80/21	0.6619 ± 0.1579	0.6168 ± 0.2000	0.6381 ± 0.2074	0.7129 ± 0.1371
Paclitaxel	2,281/570	0.7903 ± 0.0227	0.7573 ± 0.0334	0.8919 ± 0.0168	0.8423 ± 0.0149
Paclitaxel–Atezolizumab	3,690/922	0.7550 ± 0.0615	0.7034 ± 0.0984	0.8817 ± 0.0550	0.8595 ± 0.0663
Cisplatin	343/86	0.6860 ± 0.0588	0.6309 ± 0.0836	0.7411 ± 0.0645	0.8161 ± 0.0386

As shown in Table S7, CSGDA retained predictive ability on target cells excluded from adaptation. The three larger datasets achieved held-out AUC values of 0.9983, 0.8919, and 0.8817, whereas the small PLX4720-A375 dataset showed lower and more variable performance because its test subset contained only 21 cells. Overall, these results demonstrate that CSGDA retains predictive ability when the test cells are excluded from domain adaptation, providing a more conservative assessment of its generalization to previously unseen target cells.

Appendix F Supplementary Paradigm Validation Analysis

To comprehensively evaluate the robustness of our architecture, we further assessed CSGDA and six baseline methods under the traditional Bulk-to-Single-Cell (Bulk-to-SC) transfer paradigm. In this setting, all models were trained exclusively on bulk RNA-seq source data and evaluated on scRNA-seq target data. As presented in Table S8, traditional machine learning algorithms (e.g., AdaBoost, SVM) and several deep learning baselines struggle significantly due to the severe manifold structure mismatches inherent in this cross-modal transfer. Despite these challenging non-I.I.D. shifts, CSGDA consistently achieves superior and stable predictive performance across all experimental scenarios. The corresponding means and standard deviations of ACC, AUC, and AUPR are summarized in Table S9. This demonstrates that the architectural innovations of CSGDA—particularly the cell state-guided graph learning and overlap-penalized domain adaptation—possess intrinsic robustness and generalization capabilities, even when limited to traditional bulk source domains. The detailed descriptions of the source and target datasets used for this Bulk-to-SC baseline evaluation are summarized in **Table S3** and **Table S4**, respectively.

Controlled Paradigm Comparison. To isolate the effect of the transfer paradigm from that of the model architecture, we evaluated the same CSGDA architecture under Bulk-to-SC and SC-to-SC settings. Dataset availability limited this controlled comparison to two tasks. Among the datasets used in this study, only PLX4720 and Paclitaxel provided compatible bulk and single-cell source data for the same drug that could be evaluated using the same single-cell target dataset. The target dataset and training protocol were kept unchanged, while only the source modality was changed.

As shown in Table S10, SC-to-SC transfer increased AUPR from 0.6801 to 0.8850 in the heterogeneous Paclitaxel cross-tissue task. In contrast, both paradigms achieved near-saturated performance in the PLX4720 cross-platform task. These results indicate that the benefit of SC-to-SC transfer is context dependent and is most evident when the source and target domains differ substantially in biological composition.

Table S8: Performance comparison of computational models across diverse datasets

Method	Gefitinib			Afatinib			AR-42			Cetuximab		
	ACC	AUC	AUPR	ACC	AUC	AUPR	ACC	AUC	AUPR	ACC	AUC	AUPR
AdaBoost	0.485	0.370	0.435	0.467	0.492	0.547	0.470	0.264	0.373	0.525	0.572	0.603
SVM	0.530	0.466	0.526	0.533	0.512	0.581	0.485	0.600	0.602	0.467	0.460	0.501
scDEAL	0.500	0.683	0.708	0.467	0.353	0.157	0.500	0.531	0.370	0.500	0.468	0.447
SCAD	-	0.967	0.973	-	0.880	0.889	-	0.968	0.970	-	0.923	0.921
scAdadrug	0.800	0.840	0.878	0.583	0.725	0.720	0.500	0.550	0.541	0.534	0.798	0.784
scGSDR	0.769	0.952	0.948	0.833	0.923	0.920	0.786	0.896	0.938	0.833	0.871	0.833
CSGDA	**1.000**	**1.000**	**1.000**	**0.992**	**1.000**	**1.000**	**0.985**	**1.000**	**1.000**	**0.942**	**0.982**	**0.982**

Method	Etoposide			NVP-TAE684			Sorafenib			Vorinostat		
	ACC	AUC	AUPR	ACC	AUC	AUPR	ACC	AUC	AUPR	ACC	AUC	AUPR
AdaBoost	0.548	0.501	0.562	0.475	0.489	0.496	0.475	0.489	0.522	0.576	0.676	0.676
SVM	0.447	0.590	0.559	0.500	0.458	0.502	0.525	0.594	0.600	0.500	0.615	0.632
scDEAL	0.452	0.476	0.407	0.500	0.698	0.744	0.500	0.568	0.558	0.500	0.486	0.283
SCAD	-	0.694	0.736	-	0.598	0.664	-	0.582	0.627	-	0.902	0.904
scAdadrug	0.607	0.629	0.669	0.437	0.734	0.821	0.388	0.339	0.420	0.550	0.590	0.638
scGSDR	0.595	0.652	0.633	0.792	0.821	0.868	0.833	0.832	0.890	0.769	0.929	0.959
CSGDA	**0.773**	**0.870**	**0.908**	**0.958**	**0.969**	**0.969**	**0.933**	**0.975**	**0.981**	**0.970**	**0.996**	**0.997**

Table S9: Performance across seven drug-specific Bulk-to-SC tasks (Gefitinib, Afatinib, AR-42, Cetuximab, NVP-TAE684, Sorafenib, and Vorinostat)

Method	ACC	AUC	AUPR
AdaBoost	0.4961 ± 0.0403	0.4789 ± 0.1329	0.5217 ± 0.1011
SVM	0.5057 ± 0.0248	0.5293 ± 0.0716	0.5634 ± 0.0531
scDEAL	0.4953 ± 0.0125	0.5410 ± 0.1220	0.4667 ± 0.2171
scAdaDrug	0.5417 ± 0.1322	0.6537 ± 0.1736	0.6860 ± 0.1633
SCAD	–	0.8314 ± 0.1681	0.8497 ± 0.1434
scGSDR	0.8021 ± 0.0300	0.8891 ± 0.0500	0.9080 ± 0.0462
**CSGDA**	$\mathbf{0.9686 \pm 0.0255}$	$\mathbf{0.9889 \pm 0.0133}$	$\mathbf{0.9899 \pm 0.0125}$

Table S10: Paradigm-level comparison using the same CSGDA architecture

Experiment	Transfer scenario	Paradigm	ACC	Macro-F1	AUROC	AUPR
Exp. 3: Paclitaxel	Cross-tissue	Bulk-to-SC	0.7455	0.7384	0.8026	0.6801
Exp. 3: Paclitaxel	Cross-tissue	SC-to-SC	0.8576	0.8462	0.9245	0.8850
Exp. 1: PLX4720	Cross-platform	Bulk-to-SC	0.9971	0.9971	0.9969	0.9962
Exp. 1: PLX4720	Cross-platform	SC-to-SC	0.9947	0.9947	0.9999	0.9999

Note: The CSGDA architecture, target dataset, and training protocol were kept unchanged between the two paradigms. Only the source modality was changed from bulk RNA-seq to scRNA-seq.

Appendix G Implementation Details

Computational Environment. All experiments were conducted on a workstation running Ubuntu 22.04 LTS, equipped with an Intel Xeon 16-core CPU, 128 GB RAM, and a single NVIDIA GeForce RTX 3090 GPU (24 GB VRAM). The software environment included Python 3.10, PyTorch 2.4.1, DGL 2.4.0, and PyTorch Geometric 2.6.1.

Hyperparameter Selection. Graph-related parameters were initialized using the established CSA-GSL configuration and verified in preliminary experiments. The remaining parameters were selected using source-domain validation without target response labels, and the resulting configuration was fixed across all five SC-to-SC tasks.

CSA-GSL Configuration. The module operates independently on source and target domains based on the ProGNN framework. We initialize affinity graphs using KNN ($k = 15$) on the cell state matrix X. Optimization follows an alternating scheme: 2 inner steps for structure updates (G) and 1 outer step for GCN parameter updates (θ) per epoch. For convergence monitoring, an 80/20 data split is applied independently to the source and target graphs, stratified by the expression-derived cell-state pseudo labels. No drug-response labels are used in this split. For each domain, the refined graph yielding the highest validation accuracy on the held-out 20% pseudo-label classification subset is selected for downstream representation learning. The backbone is a GCN with a hidden dimension of 16 and a dropout rate of 0.6.

G.1 Detailed CSA-GSL Optimization

Initial graph construction. Using the cell-state score matrix X, we construct the initial cell affinity graph $\mathcal{G}_{\text{init}} = (A, X)$ using KNN. The neighborhood $\mathcal{N}_k(v_i)$ of cell i is determined by cosine similarity between cell-state score vectors. The binary adjacency matrix $A \in \{0,1\}^{N \times N}$ is defined as

$$A_{ij} = \begin{cases} 1, & \text{if } v_j \in \mathcal{N}_k(v_i) \text{ or } v_i \in \mathcal{N}_k(v_j), \\ 0, & \text{otherwise.} \end{cases} \tag{1}$$

Although constructed from biologically informed state scores, $\mathcal{G}_{\text{init}}$ may retain spurious connections caused by technical noise and therefore serves as the initial graph for subsequent CSA-GSL refinement.

Graph regularization terms. The CSA-GSL objective combines pseudo-label classification, graph fidelity, sparsity, low-rank, and feature-smoothness terms, weighted by γ, ϕ, α, β, and λ, respectively. The fidelity term $\phi\|G - A\|_F^2$ limits excessive deviation from the initial KNN topology. The sparsity term $\alpha\|G\|_1$ suppresses weak edges, whereas the nuclear-norm term $\beta\|G\|_*$ regularizes the rank of the refined graph. The feature-smoothness term $\lambda \operatorname{tr}(X^T \hat{L}_G X)$ encourages connected cells to have similar state-score profiles.

For the refined weighted graph G, the degree matrix $D \in \mathbb{R}^{N \times N}$ and normalized graph Laplacian $\hat{L}_G$ are defined as

$$D_{ii} = \sum_{j=1}^{N} G_{ij}, \qquad \hat{L}_G = D^{-1/2}(D - G)D^{-1/2}. \tag{2}$$

Here, D is diagonal, and D_{ii} denotes the weighted degree of cell i, calculated as the sum of its incident edge weights. For a symmetric graph, the feature-smoothness term can equivalently be written as

$$\operatorname{tr}(X^T \hat{L}_G X) = \frac{1}{2} \sum_{i,j=1}^{N} G_{ij} \left\| \frac{x_i}{\sqrt{D_{ii}}} - \frac{x_j}{\sqrt{D_{jj}}} \right\|_2^2, \tag{3}$$

where $x_i \in \mathbb{R}^8$ is the functional-state score vector of cell i. This term discourages large edge weights between cells with dissimilar normalized state profiles while accounting for differences in node connectivity.

Starting from $\mathcal{G}_{\text{init}}$, CSA-GSL optimizes the graph structure G and GNN parameters θ in an alternating manner. With θ fixed, G is updated by Forward-Backward Splitting to handle the differentiable and non-smooth terms in $\mathcal{L}_{\text{GSL}}$. With G fixed, θ is updated by standard gradient descent.

a. Graph structure update. We first apply gradient descent to the differentiable terms:

$$G_{\text{gd}}^{(k)} = G^{(k-1)} - \eta_G \nabla_G \Big[\phi \|A - G\|_F^2 + \lambda \operatorname{tr}(X^T \hat{L}_G X) + \gamma \mathcal{L}_{\text{GNN}}(\theta, G, X, \mathcal{Y}_L) \Big], \tag{4}$$

where k denotes the iteration index and $\eta_G = 0.01$ is the graph learning rate.

To enforce the low-rank constraint, we apply the proximal operator of the nuclear norm. Given the singular value decomposition

$$G_{\text{gd}}^{(k)} = U \operatorname{diag}(\sigma_i) V^T, \tag{5}$$

the low-rank update is

$$G_{\text{lr}}^{(k)} = U \operatorname{diag}\left[(\sigma_i - \eta_G \beta)_+\right] V^T, \tag{6}$$

where $(a)_+ = \max(a, 0)$.

The proximal operator of the L_1 norm is subsequently applied to enforce sparsity:

$$G_{\text{sp}}^{(k)} = \operatorname{sgn}\left(G_{\text{lr}}^{(k)}\right) \odot \left(\left|G_{\text{lr}}^{(k)}\right| - \eta_G \alpha\right)_+, \tag{7}$$

where $\odot$ denotes the Hadamard product. This soft-thresholding operation removes edges with low weights.

Finally, the graph is projected onto the set of symmetric matrices with edge weights in $[0, 1]$:

$$G^{(k)} = \max\left[0, \min\left(1, \frac{G_{\text{sp}}^{(k)} + \left(G_{\text{sp}}^{(k)}\right)^T}{2}\right)\right]. \tag{8}$$

b. GNN parameter update. With the updated graph $G^{(k)}$ fixed, the GNN parameters are updated as

$$\theta^{(k+1)} = \theta^{(k)} - \eta_\theta \nabla_\theta \mathcal{L}_{\text{GNN}}\left(\theta^{(k)}, G^{(k)}, X, \mathcal{Y}_L\right), \tag{9}$$

where η_θ is the GNN learning rate and $\mathcal{Y}_L$ denotes the expression-derived cell-state pseudo labels. The graph and GNN updates are alternated until convergence, yielding the refined graph used by the shared Graph Transformer encoder.

G.2 Detailed Shared Graph Transformer Operations

The initial KNN graphs are refined separately for the source and target domains by CSA-GSL before downstream model training. At each training epoch, the complete expression matrices and refined adjacency matrices of both domains are processed by the same Graph Transformer encoder.

Input feature projection. Because X denotes the cell-state score matrix in CSA-GSL, we use e_i to denote the raw gene expression vector of cell i. Before graph-based attention, each expression vector is projected into a continuous latent feature space:

$$h_i^{(0)} = W_{\text{in}} e_i + b_{\text{in}}, \tag{10}$$

where $e_i \in \mathbb{R}^M$ is the gene expression vector, $W_{\text{in}} \in \mathbb{R}^{d \times M}$ is the projection matrix, and $b_{\text{in}} \in \mathbb{R}^d$ is the bias vector.

Graph-constrained multi-head attention. At Graph Transformer layer l, the query, key, and value vectors of attention head m are computed as

$$q_i^m = H_i^{(l)} W_Q^m, \qquad k_j^m = H_j^{(l)} W_K^m, \qquad v_j^m = H_j^{(l)} W_V^m. \tag{11}$$

The attention calculation is restricted to the refined graph neighborhood $\mathcal{N}_i$:

$$\alpha_{ij}^m = \frac{\exp\left[G_{ij}(q_i^m)^T k_j^m/\sqrt{d_m}\right]}{\sum_{r\in\mathcal{N}_i}\exp\left[G_{ir}(q_i^m)^T k_r^m/\sqrt{d_m}\right]}, \qquad j \in \mathcal{N}_i, \tag{12}$$

and $\alpha_{ij}^m = 0$ for $j \notin \mathcal{N}_i$. Here, W_Q^m, W_K^m, and W_V^m are learnable projection matrices, d_m is the dimensionality of each attention head, and G_{ij} modulates the attention score according to the refined edge weight. Restricting the normalization to $\mathcal{N}_i$ prevents aggregation from cells that are not connected in the refined graph. The factor $\sqrt{d_m}$ limits the magnitude of the attention logits and stabilizes softmax optimization.

Feature aggregation and residual normalization. The values from neighboring cells are weighted by their attention coefficients, concatenated across M_h attention heads, and mapped to the output dimension:

$$\hat{h}_i = \mathrm{BN}\left[h_i^{(l)} + \left(\Big\Vert_{m=1}^{M_h}\sum_{j\in\mathcal{N}_i}\alpha_{ij}^m v_j^m\right)W_O\right], \tag{13}$$

where M_h is the number of attention heads, $\Vert$ denotes concatenation, W_O is the output projection matrix, and BN denotes batch normalization. The residual connection preserves the input representation while the graph-constrained attention aggregates information from the refined cellular neighborhood.

G.3 Gradient Reversal for Adversarial Alignment

The adversarial alignment module consists of the shared encoder E_{sh}, a domain discriminator D, and a Gradient Reversal Layer (GRL). The discriminator is trained to distinguish source embeddings from target embeddings by minimizing the domain loss defined in Eq. 7 of the main text.

The GRL is represented by a pseudo-function $R(\cdot)$. During forward propagation, it performs the identity mapping

$$R(Z) = Z. \tag{14}$$

During backpropagation, it reverses the gradient passed from the domain discriminator to the shared encoder:

$$\frac{\partial R}{\partial Z} = -I. \tag{15}$$

Consequently, the discriminator minimizes $\mathcal{L}_{\text{dom}}$, whereas the shared encoder receives the negative domain-loss gradient and is therefore optimized to increase domain confusion.

Considering the source classification loss, target entropy loss, and adversarial domain loss, the shared-encoder parameters are updated as

$$\theta_{\text{enc}} \leftarrow \theta_{\text{enc}} - \eta\left[\nabla_{\theta_{\text{enc}}}\mathcal{L}_{\text{cls}} + \lambda_{\text{e}}\nabla_{\theta_{\text{enc}}}\mathcal{L}_{\text{ent}} - \lambda_d\nabla_{\theta_{\text{enc}}}\mathcal{L}_{\text{dom}}\right], \tag{16}$$

where θ_{enc} denotes the shared-encoder parameters, η is the learning rate, λ_{e} weights target entropy minimization, and λ_d controls the strength of adversarial domain alignment. The negative sign applied to the domain-loss gradient encourages the shared encoder to reduce domain-discriminative variation, while the discriminator itself is optimized in the conventional direction to distinguish the two domains.

G.4 Loss-Weight Scheduling

The source classification loss combines cross-entropy and the overlap penalty:

$$\mathcal{L}_{\mathrm{cls}} = \lambda_{\mathrm{c}}\mathcal{L}_{\mathrm{ce}} + \lambda_{\mathrm{o}}\mathcal{L}_{\mathrm{olp}}, \tag{17}$$

where λ_{c} and λ_{o} are fixed throughout training, with $\lambda_{\mathrm{c}} = \lambda_{\mathrm{o}} = 1.0$.

To prevent premature confidence in target predictions, the entropy-minimization coefficient increases linearly with the training epoch:

$$\lambda_{\mathrm{e}}(t) = 0.1\frac{t}{T_{\max}}, \tag{18}$$

where t is the current epoch and $T_{\max}$ is the total number of training epochs.

The adversarial domain-alignment coefficient follows a dynamic warm-up schedule:

$$\lambda_d(p) = \frac{2}{1+\exp(-10p)} - 1, \tag{19}$$

where p denotes the normalized training progress. At the beginning of training, the small value of λ_d limits adversarial alignment so that the model can first learn predictive biological representations. As training proceeds, λ_d gradually increases, strengthening the domain-invariance constraint.

Model Architecture & Training. The shared encoder employs a 3-layer Graph Transformer with 4 attention heads. The hidden dimension is set to 1024, compressing to a latent dimension of 256, with a dropout of 0.5 applied to attention and projections. The model is trained using the Adam optimizer (learning rate 1×10^{-2}, weight decay 5×10^{-4}) with a full-batch strategy for a maximum of 100 epochs. Model selection is based on the source-domain validation metric. Target-domain response labels are stored separately from the training inputs and are loaded only by the final evaluation routine after all model parameters and hyperparameters have been fixed.

Computational Cost. We profiled both the offline CSA-GSL graph-refinement stage and the downstream prediction stage. Peak GPU memory denotes the maximum PyTorch-allocated memory during the corresponding stage.

The full-matrix CSA-GSL stage remained the principal computational bottleneck. For PLX4720, which contained the largest individual graph with 6,548 target cells, CSA-GSL required 4,452.27 s (approximately 1.24 h), accounting for most of the full-pipeline runtime of 4,597.82 s (approximately 1.28 h). The peak full-pipeline GPU memory was 11.469 GB. All evaluated tasks therefore remained feasible on a single 24-GB GPU, although graph-refinement time increased substantially with graph size. For larger single-cell datasets, scalability could be improved through approximate KNN construction, sparse candidate-edge optimization, graph partitioning, neighbor sampling, mini-batch subgraph training, or low-rank factorization of the adjacency matrix.

Appendix H Biological Interpretability Analysis

To substantiate the biological mechanisms discussed in the main text, this appendix provides supplementary evidence, including a detailed list of the top high-contribution genes (Table S12), their functional enrichment and gene–term relationships (Figure S4), and a heatmap of their expression patterns (Figure S5).

Functional Enrichment of High-Contribution Genes. To further assess the biological coherence of Cisplatin attribution, we analyzed high-contribution genes from the cross-metastasis experiment using gene sets from GO, KEGG, and Reactome. As shown in Figure S4, these

Table S11: Dataset scale and computational cost of the complete CSGDA pipeline

(A) Dataset scale

Dataset	Source cells	Target cells	Source retained genes	Target retained genes
PLX4720	163	6,548	12,400	14,341
PLX4720-A375	163	101	12,400	9,750
Paclitaxel	5,183	2,851	7,427	9,359
Paclitaxel–Atezolizumab	2,053	4,612	10,677	10,163
Cisplatin	611	429	22,744	22,744

(B) Runtime

Dataset	Training time 100 epochs (s)	Inference time (s)	CSA-GSL time 300 epochs (s)	Full-pipeline time (s)
PLX4720	62.21 ± 2.56	0.371 ± 0.008	4452.27	4597.82
PLX4720-A375	5.16 ± 0.04	0.0088 ± 0.0004	12.64	26.62
Paclitaxel	33.37 ± 0.03	0.0871 ± 0.0005	2242.56	2334.45
Paclitaxel–Atezolizumab	25.54 ± 0.02	0.0227 ± 0.0001	1460.56	1523.91
Cisplatin	5.06 ± 0.01	0.0102 ± 0.0001	38.71	63.56

(C) Peak GPU memory

Dataset	CSA-GSL peak GPU (GB)	Full-pipeline peak GPU (GB)
PLX4720	2.740	11.469
PLX4720-A375	0.018	0.749
Paclitaxel	1.719	9.340
Paclitaxel–Atezolizumab	1.378	6.355
Cisplatin	0.041	1.085

Table S12: Top 20 High-Contribution Genes Identified by CSGDA and Their Matched Biological Functional States

Rank	Gene	IG Score	Matched State	Rank	Gene	IG Score	Matched State
1	*RAD51C*	0.099	DNA Repair	11	*CRADD*	0.056	DNA Damage; Apoptosis
2	*EEF1A1*	0.090	Stemness	12	*SSRP1*	0.055	DNA Repair
3	*PAM*	0.075	Hypoxia	13	*MAD2L2*	0.055	DNA Repair; DNA Damage
4	*POLR2G*	0.066	DNA Repair	14	*GNG10*	0.055	–
5	*PSMD14*	0.064	DNA Damage	15	*UBR7*	0.054	Cell Cycle
6	*CETN2*	0.063	DNA Repair	16	*TMEM205*	0.053	–
7	*NDUFA11*	0.061	–	17	*PFKP*	0.053	Hypoxia
8	*ERCC1*	0.059	DNA Repair	18	*GRPEL1*	0.052	Cell Cycle
9	*NDUFS6*	0.058	–	19	*EXOSC1*	0.052	–
10	*MCM3*	0.058	EMT	20	*F3*	0.052	Hypoxia

genes were predominantly associated with DNA repair, double-strand-break repair, single-stranded DNA binding, and nucleotide-excision repair. Notably, the DNA-repair signal was recovered in both GO and Reactome, providing convergent support across distinct annotation resources. Cisplatin induces intra- and interstrand DNA crosslinks that interfere with replication and transcription, whereas enhanced lesion recognition and nucleotide-excision repair can promote resistance [5, 6]. Among the prioritized genes, *RAD51C* contributes to homologous-recombination repair and has been experimentally associated with cisplatin resistance [3], while the ERCC1–XPF endonuclease participates in the removal of platinum-induced DNA lesions and has been linked to cisplatin resistance in head and neck and ovarian cancers [7, 11]. Other

attributed genes, including *POLR2G*, *SSRP1*, *CETN2*, and *MAD2L2*, further connect the identified gene set to transcription-coupled repair, chromatin regulation, genome maintenance, and DNA-damage tolerance. These results suggest that CSGDA captures conserved resistance-related programs across primary and metastatic tumor sites and may help prioritize candidates for subsequent perturbation and biomarker studies. Nevertheless, these attribution results remain hypothesis-generating and require experimental and clinical validation.

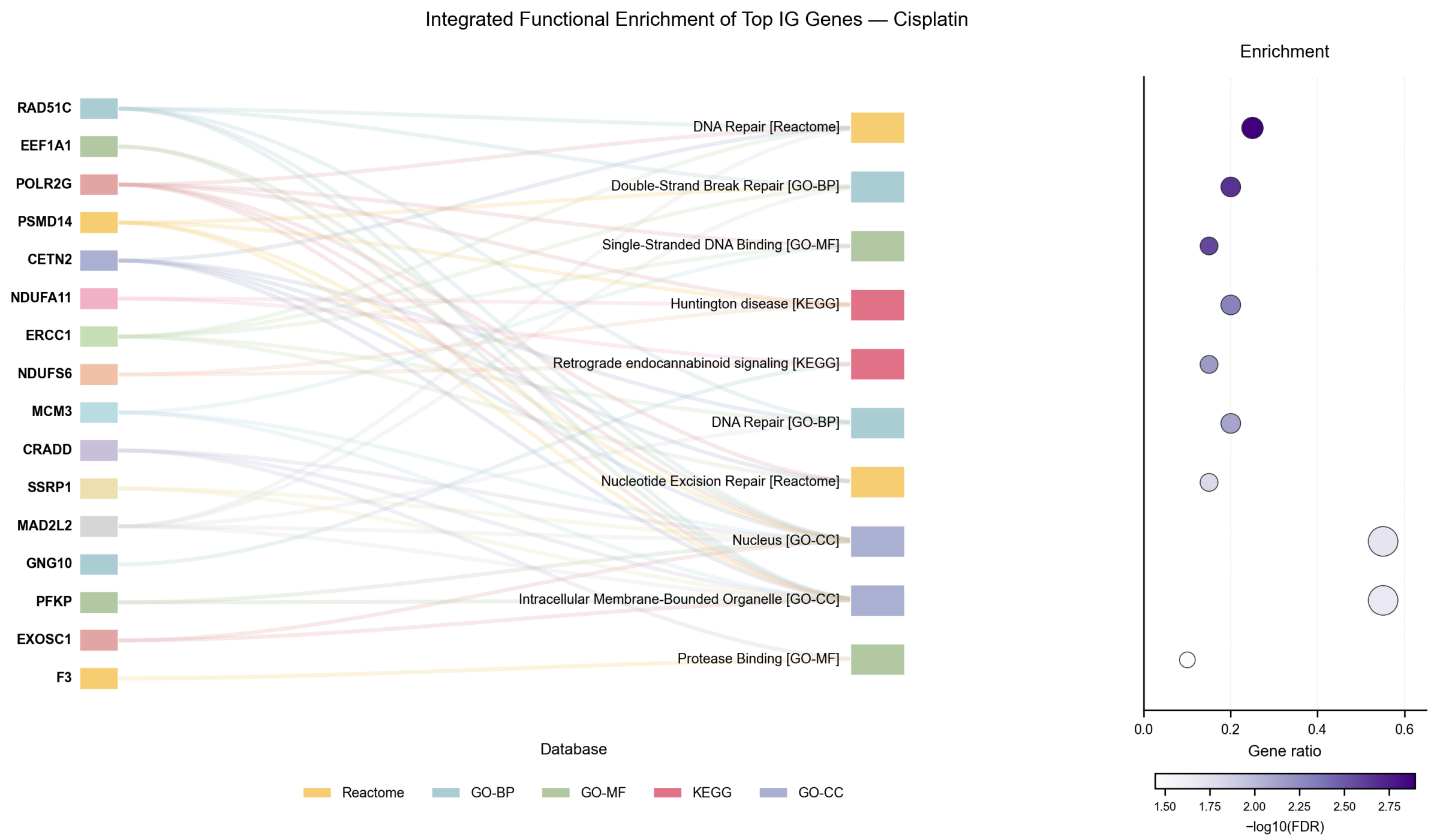

Figure S4: Integrated functional enrichment of top IG genes in the Cisplatin cross-metastasis experiment. Links connect attributed genes to their enriched functional terms. Bubble position represents the gene ratio, and color represents $-\log_{10}(\mathrm{FDR})$.

Table S13: Top 20 resistance-associated genes identified by IG in the paclitaxel cross-tissue dataset.

Rank	Gene	IG score	State	Rank	Gene	IG score	State
1	KLF6	0.061845	Hypoxia	11	GBP2	0.046127	Quiescence
2	NUTM2B-AS1	0.058314	–	12	ISG15	0.045752	–
3	NAPRT	0.058042	–	13	POLR2G	0.044155	DNA repair
4	TIMP1	0.055811	EMT	14	NPM1	0.042682	DNA damage
5	LGALS9	0.054937	Apoptosis	15	SHISA5	0.041546	Apoptosis
6	IFI6	0.051678	–	16	IRF7	0.041485	–
7	CDKN1A	0.050054	DNA damage	17	SLBP	0.039065	Cell cycle
8	SLC2A3	0.047870	Hypoxia	18	PLIN2	0.037055	Hypoxia
9	HLA-DRB5	0.047159	–	19	FOS	0.036920	Hypoxia
10	UBC	0.046673	DNA damage	20	BAX	0.036201	DNA damage

To examine whether the IG findings generalized beyond Cisplatin, we applied the same analysis to the Paclitaxel cross-tissue dataset. As shown in Table S13, the highly attributed genes implicated hypoxia, EMT, apoptosis regulation, and interferon-related stress responses. Hypoxia may reduce paclitaxel sensitivity through HIF-1α-dependent cell-cycle arrest, whereas TIMP1 may promote resistance by suppressing paclitaxel-induced apoptosis [13, 29]. The identification of IFI6, ISG15, and IRF7 further suggests that redox homeostasis, cancer stem-like properties, and inflammatory or interferon signaling contribute to resistant cell states [2, 9, 20].

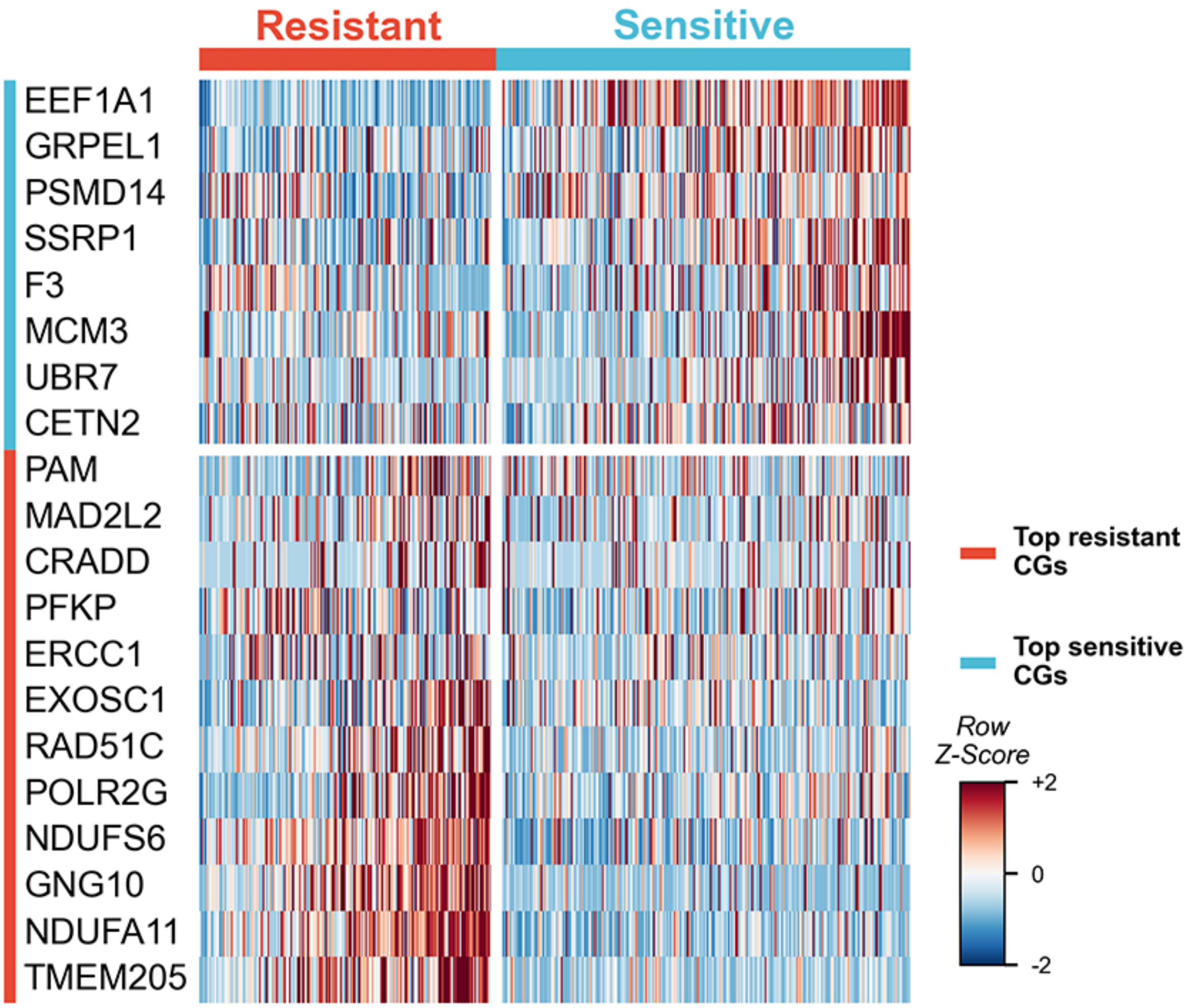

Figure S5: Heatmap displaying the relative expression levels of top predictive genes, revealing distinct expression patterns between sensitive and resistant sample clusters.

Appendix I Evaluation metric definition

The specific definitions are as follows:

- **AUROC**: Measures the classification capability of the model under various threshold settings. It reflects the probability that the model ranks a positive sample higher than a negative one and is unaffected by the absolute value of predicted probabilities, making it a universal standard for assessing the overall performance of binary classification models.
- **AUPR**: Given the significant class imbalance typically present in drug response datasets, AUPR serves as a more critical robustness metric than AUROC. It focuses on the prediction quality of positive samples and is more sensitive to the model's performance on the minority class.
- **ACC**: Represents the proportion of correctly predicted samples to the total number of samples, providing an intuitive reflection of the model's overall prediction accuracy. The calculation formula is:

$$\mathrm{ACC} = \frac{TP + TN}{TP + TN + FP + FN} \tag{20}$$

- **F1-macro Score**: Considering class imbalance, we adopted the Macro-average strategy to calculate the F1-macro score. This involves calculating the F1-score for the resistant and sensitive classes separately and then taking their arithmetic mean. This approach assigns equal weight to both resistant and sensitive classes, preventing the model from being biased towards the majority class. The formula is as follows:

$$\text{F1-macro} = \frac{1}{C}\sum_{i=1}^{C}\frac{2 \times P_i \times R_i}{P_i + R_i} \tag{21}$$

Where TP, TN, FP, and FN represent True Positives, True Negatives, False Positives, and False Negatives, respectively; P_i and R_i represent the Precision and Recall for the i-th class (Resistant or Sensitive), and C denotes the number of classes (in this study, $C = 2$).

References

[1] Chen, Junyi, Wang, Xiaoying, Ma, Anjun, Wang, Qi-En, Liu, Bingqiang, Li, Lang, Xu, Dong, & Ma, Qin. 2022. Deep transfer learning of cancer drug responses by integrating bulk and single-cell RNA-seq data. *Nature Communications*, **13**(1), 6494.

[2] Chen, Ren-Hui, Du, Yong, Han, Ping, Wang, Hong-Bo, Liang, Fa-Ya, Feng, Guo-Kai, Zhou, Ai-Jun, Cai, Mu-Yan, Zhong, Qian, Zeng, Mu-Sheng, *et al.* 2016a. ISG15 predicts poor prognosis and promotes cancer stem cell phenotype in nasopharyngeal carcinoma. *Oncotarget*, **7**(13), 16910.

[3] Chen, Xiuli, Qian, Dong, Cheng, Jingjing, Guan, Yong, Zhang, Bin, Ding, Xiaofeng, Zeng, Jing, Chen, Xi, Er, Puchun, Zhang, Furong, *et al.* 2016b. High expression of Rad51c predicts poor prognostic outcome and induces cell resistance to cisplatin and radiation in non-small cell lung cancer. *Tumor Biology*, **37**(10), 13489–13498.

[4] Cluff, Emily, Magdaleno, Carina C, Fernandez, Emyly, House, Trenton, Swaminathan, Srividya, Varadaraj, Archana, & Rajasekaran, Narendiran. 2022. Hypoxia-inducible factor-1 alpha expression is induced by IL-2 via the PI3K/mTOR pathway in hypoxic NK cells and supports effector functions in NKL cells and ex vivo expanded NK cells. *Cancer Immunology, Immunotherapy*, **71**(8), 1989–2005.

[5] Dasari, Shaloam, & Tchounwou, Paul Bernard. 2014. Cisplatin in cancer therapy: molecular mechanisms of action. *European journal of pharmacology*, **740**, 364–378.

[6] Duan, Mingrui, Ulibarri, Jenna, Liu, Ke Jian, & Mao, Peng. 2020. Role of nucleotide excision repair in cisplatin resistance. *International journal of molecular sciences*, **21**(23), 9248.

[7] Ferry, Katherine V, Hamilton, Thomas C, & Johnson, Steven W. 2000. Increased nucleotide excision repair in cisplatin-resistant ovarian cancer cells: Role of ercc1–xpf. *Biochemical pharmacology*, **60**(9), 1305–1313.

[8] Friend, Creighton, Parajuli, Parash, Razzaque, Mohammed S, & Atfi, Azeddine. 2023. Deciphering epithelial-to-mesenchymal transition in pancreatic cancer. *Advances in Cancer Research*, **159**, 37–73.

[9] Gao, Wei, Sun, Linlin, Gai, Jinwei, Cao, Yinan, & Zhang, Shuqun. 2024. Exploring the resistance mechanism of triple-negative breast cancer to paclitaxel through the scRNA-seq analysis. *PLoS One*, **19**(1), e0297260.

[10] Ho, Yu-Jui, Anaparthy, Naishitha, Molik, David, Mathew, Grinu, Aicher, Toby, Patel, Ami, Hicks, James, & Hammell, Molly Gale. 2018. Single-cell RNA-seq analysis identifies markers of resistance to targeted BRAF inhibitors in melanoma cell populations. *Genome research*, **28**(9), 1353–1363.

[11] Hsu, Dennis Shin-Shian, Lan, Hsin-Yi, Huang, Chi-Hung, Tai, Shyh-Kuan, Chang, Shyue-Yih, Tsai, Tung-Lung, Chang, Cheng-Chi, Tzeng, Cheng-Hwai, Wu, Kou-Juey, Kao, Jung-Yie, *et al.* 2010. Regulation of excision repair cross-complementation group 1 by Snail contributes to cisplatin resistance in head and neck cancer. *Clinical Cancer Research*, **16**(18), 4561–4571.

[12] Huang, Kaishun, & Liu, Hui. 2025. Identification of drug-resistant individual cells within tumors by semi-supervised transfer learning from bulk to single-cell transcriptome. *Communications Biology*, **8**(1), 530.

[13] Huang, Lei, Ao, Qilin, Zhang, Qinghua, Yang, Xiaokui, Xing, Hui, Li, Fang, Chen, Gang, Zhou, Jianfeng, Wang, Shixuan, Xu, Gang, *et al.* 2010. Hypoxia induced paclitaxel resistance in human ovarian cancers via hypoxia-inducible factor 1α. *Journal of cancer research and clinical oncology*, **136**(3), 447–456.

[14] Huang, Yu-An, Cao, Xiyue, You, Zhu-Hong, Li, Yue-Chao, Shang, Xuequn, & Huang, Zhi-An. 2025. scGSDR: Harnessing gene semantics for single-cell pharmacological profiling. *Communications Biology*, **8**(1), 1508.

[15] Kais, Zeina, Rondinelli, Beatrice, Holmes, Amie, O'Leary, Colin, Kozono, David, D'Andrea, Alan D, & Ceccaldi, Raphael. 2016. FANCD2 maintains fork stability in BRCA1/2-deficient tumors and promotes alternative end-joining DNA repair. *Cell reports*, **15**(11), 2488–2499.

[16] Li, Yan-Ruide, Halladay, Tyler, & Yang, Lili. 2024. Immune evasion in cell-based immunotherapy: unraveling challenges and novel strategies. *Journal of Biomedical Science*, **31**(1), 5.

[17] Lindell, Emma, Zhong, Lei, & Zhang, Xiaonan. 2023. Quiescent cancer cells—a potential therapeutic target to overcome tumor resistance and relapse. *International journal of molecular sciences*, **24**(4), 3762.

[18] Liu, Hui, Duan, Wei, & Luo, Judong. 2025. Predicting Single-Cell Drug Sensitivity Utilizing Adaptive Weighted Features for Multi-Source Domain Adaptation. *IEEE Journal of Biomedical and Health Informatics*, **29**(8), 6043–6054.

[19] Liu, Xiaona, Yi, Jiahao, Li, Tina, Wen, Jianguo, Huang, Kexin, Liu, Jiajia, Wang, Grant, Kim, Pora, Song, Qianqian, & Zhou, Xiaobo. 2024. DRMref: comprehensive reference map of drug resistance mechanisms in human cancer. *Nucleic acids research*, **52**(D1), D1253–D1264.

[20] Liu, Zhenchuan, Gu, Shaorui, Lu, Tiancheng, Wu, Kaiqing, Li, Lei, Dong, Chenglai, & Zhou, Yongxin. 2020. IFI6 depletion inhibits esophageal squamous cell carcinoma progression through reactive oxygen species accumulation via mitochondrial dysfunction and endoplasmic reticulum stress. *Journal of Experimental & Clinical Cancer Research*, **39**(1), 144.

[21] Mao, Peng, & Wyrick, John J. 2019. Organization of DNA damage, excision repair, and mutagenesis in chromatin: A genomic perspective. *DNA repair*, **81**, 102645.

[22] Pastushenko, Ievgenia, Brisebarre, Audrey, Sifrim, Alejandro, Fioramonti, Marco, Revenco, Tatiana, Boumahdi, Soufiane, Van Keymeulen, Alexandra, Brown, Daniel, Moers, Virginie, Lemaire, Sophie, *et al.* 2018. Identification of the tumour transition states occurring during EMT. *Nature*, **556**(7702), 463–468.

[23] Rankin, Erinn B, & Giaccia, Amato J. 2016. Hypoxic control of metastasis. *Science*, **352**(6282), 175–180.

[24] Roy, Sayoni, Sunkara, Raghava R, Parmar, Manan Y, Shaikh, Saima, & Waghmare, Sanjeev K. 2020. EMT imparts cancer stemness and plasticity: new perspectives and therapeutic potential. *Frontiers in Bioscience-Landmark*, **26**(2), 238–265.

[25] Russo, Mariangela, Chen, Mengnuo, Mariella, Elisa, Peng, Haoning, Rehman, Sumaiyah K, Sancho, Elena, Sogari, Alberto, Toh, Tzen S, Balaban, Nathalie Q, Batlle, Eduard, *et al.* 2024. Cancer drug-tolerant persister cells: from biological questions to clinical opportunities. *Nature Reviews Cancer*, **24**(10), 694–717.

[26] Yang, Seungyeon, Hwang, Sunsook, Kim, Byungjoo, Shin, Seungmin, Kim, Minjoong, & Jeong, Seung Min. 2023. Fatty acid oxidation facilitates DNA double-strand break repair by promoting PARP1 acetylation. *Cell Death & Disease*, **14**(7), 435.

[27] Zhang, Yuanyuan, Chen, Hongyan, Mo, Hongnan, Hu, Xueda, Gao, Ranran, Zhao, Yahui, Liu, Baolin, Niu, Lijuan, Sun, Xiaoying, Yu, Xiao, *et al.* 2021. Single-cell analyses reveal key immune cell subsets associated with response to PD-L1 blockade in triple-negative breast cancer. *Cancer cell*, **39**(12), 1578–1593.

[28] Zheng, Zetian, Chen, Junyi, Chen, Xingjian, Huang, Lei, Xie, Weidun, Lin, Qiuzhen, Li, Xiangtao, & Wong, Ka-Chun. 2023. Enabling single-cell drug response annotations from bulk RNA-Seq using SCAD. *Advanced Science*, **10**(11), 2204113.

[29] Zhu, Dongliang, Zha, Xiaoming, Hu, Meiling, Tao, Aidi, Zhou, Hangbo, Zhou, Xiaojun, & Sun, Yujie. 2012. High expression of TIMP-1 in human breast cancer tissues is a predictive of resistance to paclitaxel-based chemotherapy. *Medical oncology*, **29**(5), 3207–3215.
